# Solvation Structure and Interface Engineering Synergy in Low-Temperature Sodium-Ion Batteries: Advances and Prospects

**DOI:** 10.3390/nano15110820

**Published:** 2025-05-29

**Authors:** Shengchen Huang, Lin Liu, Chenchen Han, Chao Tian, Yongjian Wang, Tianlin Li, Danyang Zhao, Yanwei Sui

**Affiliations:** 1 School of Materials Science and Physics, China University of Mining and Technology, Xuzhou 221116, China; ts24180096p31@cumt.edu.cn (S.H.); 13653758296@163.com (C.H.); ltl1943@outlook.com (T.L.); 2 Jiangsu Province Engineering Laboratory of High-Efficient Energy Storage Technology and Equipment, School of Materials Science and Physics, China University of Mining and Technology, Xuzhou 221116, China

**Keywords:** sodium-ion batteries, extreme low temperature, failure mechanisms, multi-scale optimized strategies, interfacial kinetics

## Abstract

The performance degradation of sodium-ion batteries (SIBs) in extremely low-temperature conditions has faced significant challenges for energy storage applications in extreme environments. This review systematically establishes failure mechanisms that govern the performance of low-temperature SIBs, including significantly increased electrolyte viscosity, lattice distortion and adverse phase transitions in electrodes, and sluggish desolvation kinetics at the solid electrolyte interface. Herein, we specifically summarize a series of multi-scale optimization strategies to address these low-temperature challenges: (1) optimizing low-freezing-point solvent components and regulating solvation structures to increase ionic diffusion conductivity; (2) enhancing the hierarchical structure of electrodes and optimizing electron distribution density to improve structural stability and capacity retention at low temperatures; and (3) constructing an inorganic-rich solid electrolyte interphase to induce uniform ion deposition, reduce the desolvation barrier, and inhibit side reactions. This review provides a comprehensive overview of low-temperature SIB applications coupled with advanced characterization and first-principles simulations. Furthermore, we highlight solvation-shell dynamics, charge transfer kinetics, and metastable-phase evolution at the atomic scale, along with the critical pathways for overcoming low-temperature limitations. This review aims to establish fundamental principles and technological guidelines for deploying advanced SIBs in extreme low-temperature environments.

## 1. Introduction

As global energy transitions accelerate, large-scale energy storage technologies are gaining strategic importance [1,2]. Sodium-ion batteries (SIBs) have emerged as a promising solution in this field, making full use of abundant sodium resources (2.64% of Earth’s crust abundance) and unique technical strengths. Their electrode potential (−2.71 V vs. SHE) is similar to that of lithium-ion batteries (LIBs) [3,4], while sharing the same “rocking-chair” ion diffusion mechanism [5]. This similarity allows partial reuse of LIB production equipment, cutting technology conversion costs. Notably, SIBs outperform LIBs in cold environments. Although sodium ions are slightly larger than lithium ions (0.102 nm vs. 0.076 nm), their smaller solvated size and optimized low-viscosity electrolytes enable faster ion transport below −20 °C. Combined with sodium’s lower reactivity reducing thermal runaway, ensuring enhanced safety and adaptability in cold climates.

On the contrary, although LIBs occupy a dominant position in the current energy storage field by virtue of their proven technology system [6], they face bottlenecks such as uneven distribution of lithium resources, high cost, and poor low-temperature adaptability, which make it difficult for them to satisfy the strict requirements of economy and environmental tolerance in large-scale energy storage scenarios [7]. Based on this, SIBs with significant advantages in resources, technology, performance, and safety, are fully capable of serving as an effective complement [8] and are widely used in large-scale energy storage, contributing to building a more economical, environmentally adaptable, and safe energy storage system in low-temperature conditions. It is worth mentioning that, when deployed in extreme environments such as deep-sea exploration, polar energy systems, and aerospace equipment [9,10,11], traditional energy storage devices suffer more severe challenges such as extreme low temperatures. However, even SIBs have remarkable low-temperature performance but inevitably suffer from significant performance degradation in more severe low-temperature environments (<−20 °C). This phenomenon stems from a significant increase in the macroscopic viscosity of the electrolyte system, which in turn triggers a sharp increase in impedance at the electrode/electrolyte interface; at the microscopic level, the charge transfer kinetics at the solid/liquid interface exhibits hysteresis characteristics, along with a degradation of the nanoscale structure of the Solid Electrolyte Interphase (SEI) and Chemical/Electrochemical Interface (CEI). Fundamental challenges arise from the synergistic effect of electrolyte stability degradation and electrode material phase structure decline, triggering a multiscale failure mechanism at the electrode/electrolyte interface, which ultimately leads to catastrophic capacity degradation, a shortened cycle life, and power density collapse.

SIBs are mainly composed of electrodes, electrolytes, and the interface between these components. The performance of each part directly determines the performance of the batteries. In order to improve the low-temperature performance of SIBs, the optimization of electrolyte components, the solventation structure, the electrode structure design, and the electrode/electrolyte modulation are expected to play an important role. The development of low-temperature SIBs essentially relies on the in-depth synergistic regulation of the electrolyte solvation structure, electrode material surface-interface properties, and desolvation kinetic processes. The solvated sheath structure [12] in the electrolyte, as a medium for ion transport, directly determines the migration number (t^+^) and ionic conductivity (σ) of sodium ions, and reorganization energy of solvation shell and desolvation energy barriers have a significant influence on the charge transfer reaction rate. The crystal structure of the electrode material as well as the surface interface engineering [13,14,15,16] determine the number of storage sites for sodium ions and the degree of optimization of the insertion and extraction pathways [17,18]. The electrochemical kinetics of the crucial desolvation [19] process, which acts as a bridge connecting the electrolyte to the electrode, is directly correlated to the charge transfer resistance (R_ct_), as well as the interfacial stability. At low temperatures, the ordering of solvent molecular orientation is enhanced, resulting in an increase in the desolvation barrier of solvated ions, which in turn triggers an intensification of the electrode/electrolyte interface (EEI) side reactions and the formation of a loose and porous SEI/CEI film, further deteriorating the cycling performance of the battery.

The breakthrough direction of future research should focus on building a systematic electrolyte design theory system, focusing on the following dimensions: at the basic mechanism level, it is necessary to deepen the multidimensional synergistic mechanism study of “solvation structure–electrode intrinsic properties–interfacial reaction” [20] and to systematically analyze the relationships between solvation coordination numbers, ion migration paths, and the electrode crystal structure through the linkage of theoretical calculations and in situ characterization techniques [21,22]. Additionally, it is important to establish the dynamic matching law of key parameters and establish the dynamic matching law of key parameters. At the methodological innovation level, we should break through the limitations of traditional single-component optimization and construct a universal regulation strategy based on the multidimensional parameters of solvent polarity, viscosity, and coordination ability [23]. Furthermore, developing a multiscale synergistic solvation structure control method is necessary. At the level of theoretical construction, it is urgent to promote the shift of the electrolyte design paradigm from empirical trial-and-error to theory-driven and to establish a systematic design framework across the micro-mechanism and macro-performance by integrating the whole-process prediction models of solvated structure evolution, interfacial film dynamics, and device performance [24,25,26]. This research paradigm not only provides a systematic solution for the optimization of the low-temperature performance of SIBs but also establishes a universal theoretical basis for the electrolyte design of new energy storage systems.

**Figure 1:**
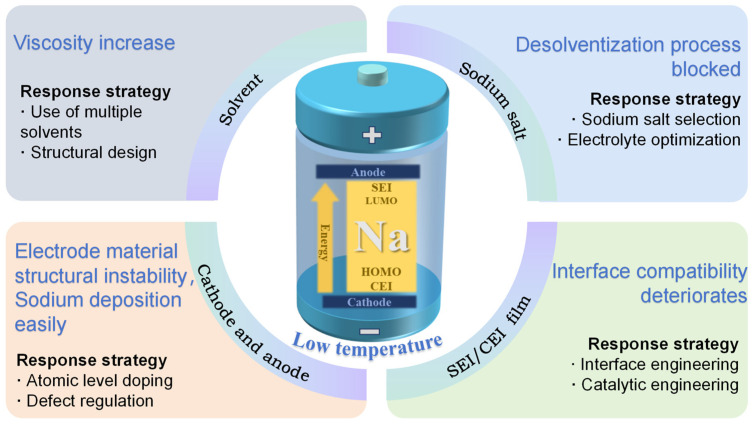
Solvation structure and interface engineering synergy in low-temperature sodium-ion batteries: advances and prospects.

This review focuses on the key scientific issues of SIBs in extreme low-temperature environments, thoroughly investigates the failure mechanisms and reveals the intrinsic correlation mechanisms, and puts forward a targeted regulation strategy to carry out a systematic review (Figure 1), aiming to provide theoretical support and a technical pathway for the development of high-performance batteries. This is of great significance to promoting the technological breakthrough of SIBs within the field of large-scale energy storage.

## 2. Failure Mechanisms

Under low-temperature conditions, the performance decline and even failure of SIBs result from the combined effects of multiple factors. At low temperatures, the viscosity of the electrolytes increases, which reduces their ionic conductivity. As a result, the transfer rate of sodium ions slows down, making the desolvation process difficult and weakening the battery’s kinetic performance. Secondly, electrolyte freezing or salt precipitation worsens battery performance. Additionally, for electrode materials at low temperatures, the ionic diffusion rate in cathode materials slows down, affecting their structural stability. Anode materials tend to experience issues like sodium deposition and unstable interface films. These problems break the electrical contact between internal components/structures of the battery, leading to performance degradation. The structure of the direct interface between electrodes and electrolytes also plays a key role. Under low-temperature conditions, the compatibility between the electrolyte and electrode materials decreases, increasing charge transfer resistance, which leads to severe polarization during charging/discharging, harming its efficiency and cycle stability, and ultimately reducing its capacity. The detailed failure mechanisms are summarized in Table 1.

**Figure 2:**
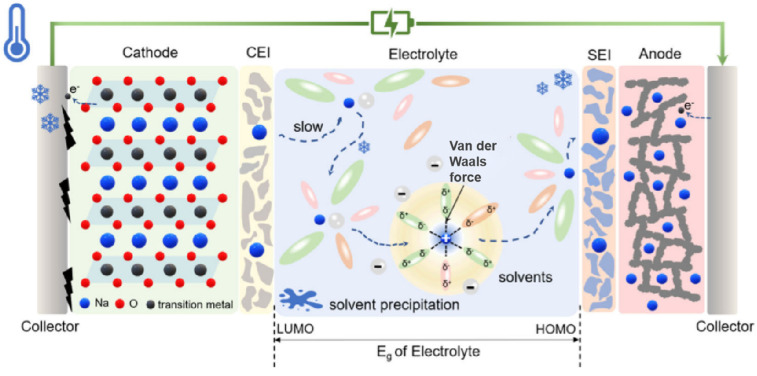
Schematic diagram of low-temperature deterioration mechanisms. Copyright 2024 Wiley [30].

The specific failure mechanism is shown in Figure 2. This process involves the diffusion of Na+ in the electrode bulk, the electrolyte, and SEI, as well as the charge transfer of Na^+^ and electrons (e^−^) at the electrode/electrolyte interface [27,28,29,30].

### 2.1. Electrolyte

#### 2.1.1. SIB Electrolyte Types and Low-Temperature Working Characteristics

The current electrolyte system for SIBs is mainly based on organic solvents, including esters and ethers. Ester electrolytes, such as ethylene carbonate (EC), propylene carbonate (PC), dimethyl carbonate (DMC), diethyl carbonate (DEC), ethyl methyl carbonate (EMC), and methyl propionate (MP), can offer advantages such as a high dielectric constant, adjustable viscosity, and specific interfacial film-forming ability, and hence have become the main commercial electrolyte for SIBs at present. During this process, molecular structures of ester solvents, including the viscosity, donor number (DN) values, the highest occupied molecular orbital (HOMO), and lowest unoccupied molecular orbital (LUMO) energy levels, significantly determine the ionic conductivity, interfacial stability, electrochemical window, and temperature adaptability of SIB electrolytes. Different ester organic solvent molecules can form clusters with unique solvation structures with sodium ions, and the sodium ion intercalation process requires a desolvation process, which is significantly influenced by the desolvation energy barriers of clusters. The properties of different ester solvent molecules are significant. Their DN values and HOMO/LUMO energy levels influence the competitive coordination ability with sodium centers and solvation structures, which can play a key role in the formation of the electrode/electrolyte interface and further determine the voltage window, cyclic performance, low-temperature properties, and stability of SIBs. Rationally blending different solvents by matching the conjugated structure, DN values, and molecular orbital can comprehensively optimize the performance of electrolytes, promoting the practical application of SIBs in terms of energy density, cycle life, and the low-temperature capacity retention ratio.

**Figure 3:**
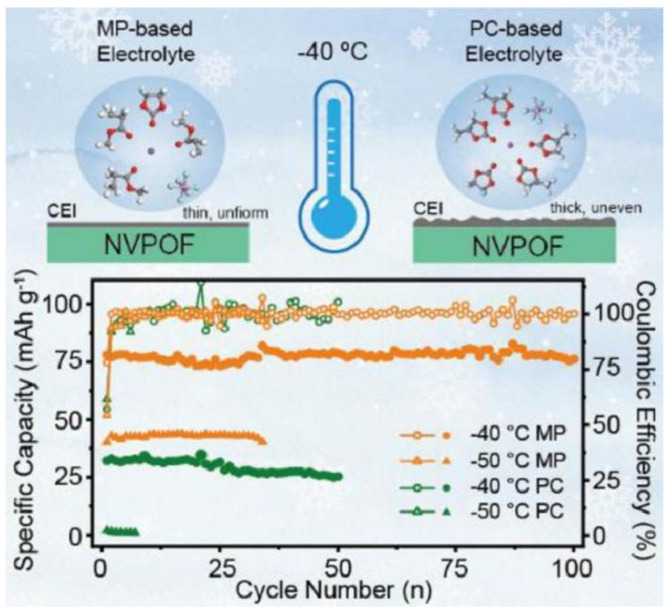
The charge/discharge mechanism and cycling performance of the Na||NVPOF battery. Copyright 2024 Elsevier [33].

Among the common carboxylic acid ester solvents, methyl propionate (MP) has a significant advantage over similar solvents due to its low viscosity, low melting point, and good compatibility with both cathode and anode materials [31,32]. Liu et al. [33] developed an MP-based electrolyte system for SIBs, which is made possible by adding fluoroethylene carbonate (FEC) as a film-forming additive. The electrolyte combines a low melting point, sufficient ionic conductivity, and high electrochemical stability under low-temperature environments, and the relevant performance indexes are significantly better than those of traditional carbonate electrolytes. When the designed carboxylate-based electrolyte is matched with the high-voltage cathode material Na_3_V_2_(PO_4_)_2_O_2_F(NVPOF), the battery system shows excellent ultra-low temperature resistance. At the extreme low temperature of −50 °C, the battery not only operates stably but also forms a robust and homogeneous CEI and maintains a high Na^+^ migration efficiency at the electrode/electrolyte interface. Due to the synergistic effect of interfacial chemistry modulation and kinetic enhancement effect, the capacity retention of the Na||NVPOF half-cell was about 89% after 500 cycles at 1 C multiplicity in a −25 °C environment, as shown in Figure 3.

Moreover, ether-based electrolytes such as tetrahydrofuran (THF) and triphenyl phosphate (TPP) have raised attention in recent years due to their low viscosity, strong Na^+^ coordination ability, and satisfactory compatibility with hard-carbon anodes. Therefore, ether-based solvents possess irreplaceable advantages in SIB systems under low-temperature conditions. In a SIB, ether solvents greatly increase ion transport efficiency, improve interface stability, and enhance cycle performance, making them more suitable for severe environments and exhibiting high energy density, large charge/discharge rates, and long cycle life at low temperatures [34,35].

**Figure 4:**
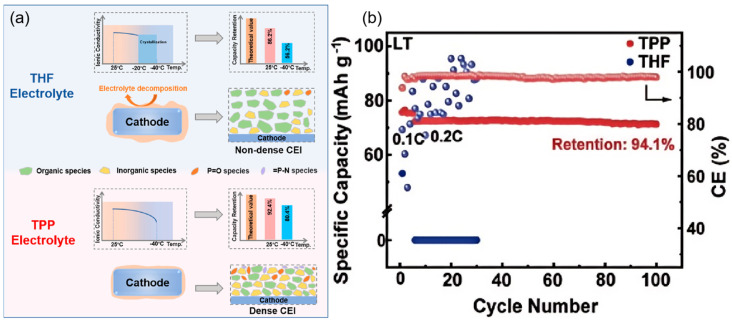
Schematic illustration of failure mechanism. (**a**) Schematic illustration of the working mechanism of selected electrolyte in the MN cathode. (**b**) Cycling performance of MN cathode in TPP and THF electrolytes at −40 °C.Copyright 2023 American Chemical Society [47].

However, practical applications of ether-based electrolyte in extreme environments are still deficient, which is caused by the problem of slow ion diffusion kinetics at low temperatures [36,37,38]. To solve this problem, researchers have tried to develop low-temperature SIB electrolytes based on mixed ether solvents due to their relatively low melting point and viscosity [39,40,41,42]. Ether solvents can be mainly classified into two types, cyclic ethers and linear ethers: linear ether solvents represented by 1,2-dimethoxyethane and diethylene glycol dimethyl ether have high chemical stability, but the strong solvation of solvent molecules with sodium ions will hinder ion transport, which will limit their performance under low-temperature conditions [43,44]. In contrast, cyclic ether solvents such as THF show better low-temperature performance due to their weaker solvation with sodium ions [45,46]. Yin et al. [47] developed a novel sodium-ion battery electrolyte with ether as the main solvent, and the assembled Na_2/3_Mn_2/3_N_i1/3_O_2_||Na battery achieved 97.2% capacity retention after 140 cycles at 25 °C and a 2.5–4.0 V voltage interval, while the THF-based electrolyte failed after only 30 cycles under the same conditions. This performance enhancement mainly originates from the CEI of phosphorus-rich and fluorine-rich components formed on the anode surface in the TPP electrolyte. When the temperature was lowered to −40 °C, the high ionic conductivity of the TPP electrolyte over a wide temperature range enabled the Na_2/3_Mn_2/3_N_i1/3_O_2_||Na battery to maintain a high capacity retention rate of 94.1% after 100 cycles (as shown in Figure 4), which demonstrates the mechanism of the two electrolytes in the Na_2/3_Mn_2/3_Ni_1/3_O_2_ positive electrode: the THF electrolyte will crystallize at −20 °C, leading to electrolyte failure, while the TPP electrolyte is able to maintain a high ionic conductivity over a wide range of temperatures, which ensures that the battery still has a high-capacity output under −40 °C. In addition, THF electrolyte continues to decompose during cycling at 25 °C, facilitating the formation of a superior CEI layer with a large number of organic components that cannot effectively protect the electrode surface; in contrast, TPP electrolyte forms a thin and dense CEI layer during cycling, with inorganic components that can stabilize the electrode interface and safeguard the efficiency of sodium ion transport.

#### 2.1.2. High Viscosity at Low Temperatures Decreases the Ionic Transport Efficiency

**Figure 5:**
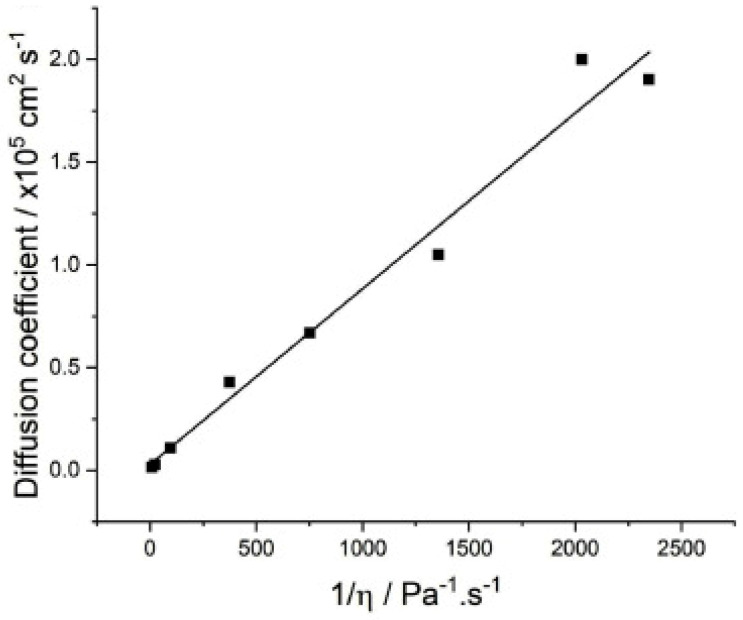
Stokes–Einstein relation with the experimental data. Copyright 2024 ELSEVIER [49].

In a low-temperature environment, the electrolyte viscosity increases significantly, which leads to a sharp decrease in the ion migration kinetics. Taking the charging process as an example, the transport of Na^+^ in the electrolyte consists of three key steps. First, the process of sodium ions undergoing a solvation process with the organic ligands in the electrolyte when embedded from the electrode. Subsequently, the solvated Na clusters undergo diffusive migration in the electrolyte. Finally, the solvated Na^+^ cluster requires a desolvation process at the surface of the electrode. During these processes, the viscosity of the electrolyte directly determines the macroscopic performance of the battery, among which ionic conductivity is an important parameter affecting the low-temperature application of SIBs. For organic solvent systems, the solvent viscosity increases sharply as the temperature drops, which is attributed to the sluggish ion diffusion conductivity for solvated clusters. To reveal quantitative relationships between the ionic diffusion coefficient, temperature, and solvent viscosity, the Stokes–Einstein equation [30] (Equation (1)) has been established as follows:

(1) $$D=\frac{kT}{6\pi \eta r}$$

where D represents the diffusion coefficient, k is the Boltzmann constant, T denotes the temperature, η is the solvent viscosity, and r is the radius of the solvated molecule. The equation shows that higher temperatures and smaller radii of solvated molecules accelerate the diffusion rate, while the opposite occurs at low temperatures [48]. In traditional electrolytes, the diffusion coefficient of ferrocene is 1.9 ± 0.2 × 10^−5^ cm^2^ s^−1^, which aligns with values reported in the literature for similar solutions (2 × 10^−5^ cm^2^ s^−1^). As the concentration approaches the high-concentration region, diffusion decreases rapidly due to the formation of contact ion pairs (CIP) and aggregate (AGG) complexes, which increases viscosity. The diffusion coefficient of Fc is plotted against fluidity in Figure 5 [49]. Logan et al. [50] evaluated electrolyte systems with the addition of a variety of ester co-solvents by measuring viscosity and ionic conductivity, using temperature as a variable, with the aim of exploring their suitability for high-rate application scenarios. The results of the study showed that regardless of the type of additives used, the electrolyte exhibited a tendency of increasing viscosity and decreasing ionic conductivity with decreasing temperature, a pattern that suggests the relative stability of the physical properties of the electrolyte during temperature changes.

#### 2.1.3. Low Temperatures Increase the Desolvation Energy Barrier

**Figure 6:**
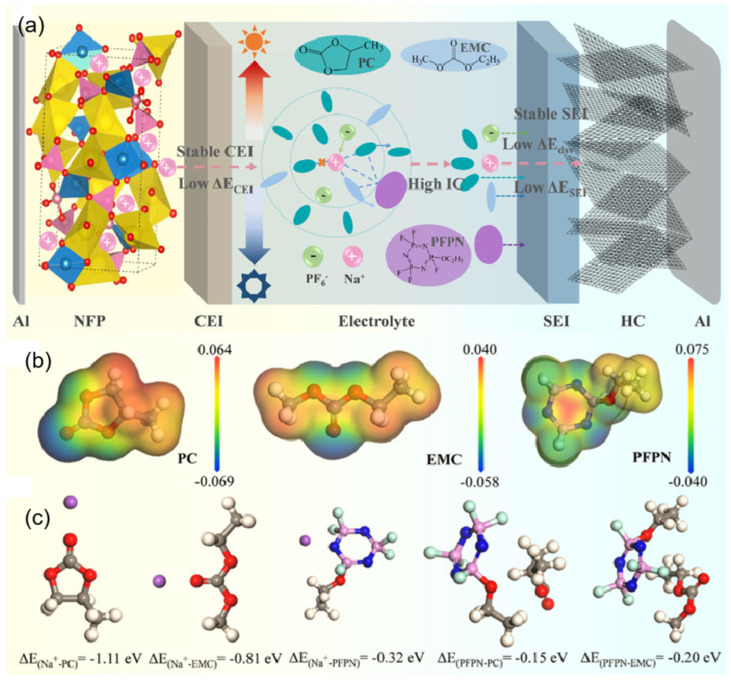
(**a**) The schematic diagram of the NFP||HC cell considers the effect of electrolytes on the transport of Na+ from cathode to anode and the formation of SEI/CEI; (**b**) ESP of PC, EMC, and PFPN; (**c**) binding energies of Na^+^-PC, Na^+^-EMC, Na^+^-PFPN, PFPN-PC, and PFPN-EMC. Copyright 2024 ELSEVIER [53].

At extremely low temperatures, the interaction between solvent molecules and sodium ions is enhanced, and the desolvation process is blocked, which makes solvent-encapsulated clusters more difficult to release Na^+^ and become embedded in the electrode, further increasing the interfacial charge transfer resistance. During the charged process, Na^+^ ions are removed from the cathode and interact with solvent molecules in the electrolyte to form a solvated Na^+^ cluster. Subsequently, they migrate to the anode surface in the presence of an electric field, exit from the solvated molecules, cross the SEI, and finally embed themselves in the anode (an anodic embedding reaction as an example) [51,52]. The low temperature leads to an increase in electrolyte viscosity and a decrease in ionic conductivity, which reduces the rate of Na^+^ shuttling through the electrolyte and makes the subsequent desolvation process difficult. This ultimately deteriorates the reaction process and leads to a severe reduction in cell kinetics [30]. Li et al. [53] used PC, which has a high dielectric constant and a wide liquid range, as the main solvent of the electrolyte, enabling the electrolyte to remain liquid under extreme conditions and contain more free ions, thus ensuring the fast migration ability of ions in the electrolyte. Then, two co-solvents with significantly different sodium ion solvation capabilities, EMC and perfluoropropyl nitrile (PFPN), were selected to synergistically regulate the first and second solvation shells of sodium ions, reconstruct the solvation structure of sodium ions, reduce the desolvation energy barrier of sodium ions, and improve the conductivity and electrode/electrolyte interface properties (Figure 6). In the theoretical calculation process, the binding energy (Δ*E_b_*) of sodium ions with different solvent molecules is calculated as follows: Δ*E_b_* = *E*_(*complex*)_ − *E*_(*A*)_ − *E*_(*Na+*)_, where *E*_(*complex*)_ is the energy of the whole system after coordination, ***E***(A) is the energy of a single molecule that constitutes the complex, and *E*_(*Na*+)_ is the energy of a single Na^+^ [54].

#### 2.1.4. The Weakening of the Entropy Effect at Low Temperatures

In recent years, regulating the solvation structure of electrolytes in SIBs by the introduction of high-entropy design strategies has emerged as an innovative direction for enhancing battery electrochemical performance. As a core parameter of the second law of thermodynamics, entropy was first proposed by Rudolf Clausius to characterize the degree of disorder in a system. It holds important physical significance in the process of energy conversion and storage. As a universal thermodynamic criterion, Gibbs free energy (∆G) can quantitatively evaluate the thermodynamic stability of any electrolyte system, and its expression is (Equation (2)):

(2) ∆G=∆H−T∆S

**Figure 7:**
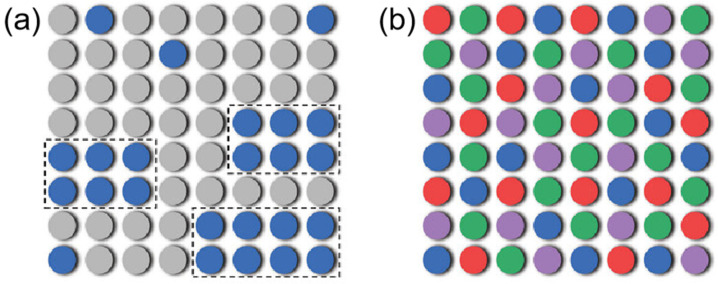
(**a**) Schematic diagram of compositional structure and energy in heterogeneous solution with precipitation of a component due to a limited solubility. (**b**) Schematic diagram of compositional structure and energy in a homogeneous solution by mixing more components to increase the entropy of mixing. Copyright 2023 Springer Nature [56].

In the equation, ∆H is the enthalpy change, T is the temperature, and ∆S is the entropy change. For electrolytes, entropy mainly refers to configurational entropy ($S_{conf}$), where the introduction of multiple components increases local disorder, thereby enhancing $S_{conf}$ (Equation (3)):

(3) $$S_{conf}=-R\sum x_{i\ln x_{i}}$$

where R is the ideal gas constant and $x_{i}$ is the mole fraction of the i-th component. The increase in Scoff enhances the mobility of ions, enabling them to rapidly adapt to changes in the electric field, thereby accelerating the rate of ion transport. In conventional electrolytes with a small number of components, the solubility of the solute is determined by the competition between ∆H (a positive value leads to lower solubility) and $S_{conf}$ (a larger value increases solubility) [55]. When the solubility limit is exceeded under specific conditions, the solute precipitates, forming a heterogeneous system, which conceptually resembles spinodal decomposition in solids (Figure 7a). Therefore, introducing multiple components to increase the mixing entropy ($S_{conf}$) in principle represents a strategy to promote the formation of a homogeneous liquid solution (Figure 7b) [56].

Therefore, ∆S can serve as a tool to analyze specific solvation structures in electrolytes. The exploration of high-entropy electrolytes has opened a new pathway for rational electrolyte design. Multi-component electrolytes offer significant disorder and diverse solvation configurations, substantially enhancing battery performance. For instance, Yang’s team [55] demonstrated that adjusting strong solvation interactions in SIBs enables spontaneous structural reorganization of solvation configurations under low-temperature conditions, thereby avoiding salt precipitation. High-performance electrolytes generally exhibit exceptional chemical/electrochemical stability and stable ion transport capabilities. Notably, binary electrolytes often display higher ionic conductivity compared to single-component systems.

### 2.2. Sodium Salts Precipitation at Low Temperature

At low temperatures, some sodium salts show a decrease in the degree of dissociation. The low-temperature environment reduces the solubility of sodium salts, which makes the high-concentration electrolyte prone to salt precipitation. Adjusting the concentration of sodium salts in the electrolyte can optimize its stability and electrochemical performance at low temperatures. To increase the concentration of sodium salts can reduce the viscosity and freezing point of the electrolyte. However, an excessively high concentration of sodium salts will lead to the precipitation of an excessive amount of crystals at low temperatures, which will damage the contact between the interfaces in the battery and further cause the decline of the battery capacity or even lead to its failure.

**Figure 8:**
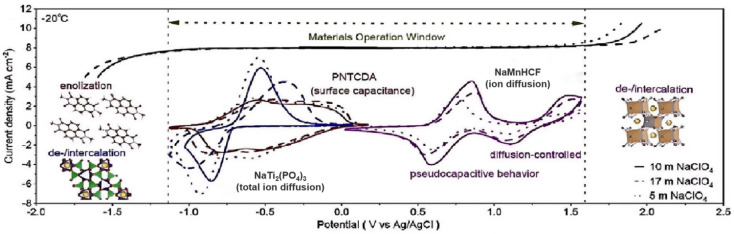
Comparison of different cathode/electrolyte battery systems. Copyright 2024 American Chemical Society [57].

For instance, for the most commonly used sodium salt electrolyte, sodium perchlorate (NaClO_4_), its freezing point decreases significantly as the concentration of sodium perchlorate increases. Han et al. [57] assessed electrode/electrolyte compatibility; we measured the electrochemical stability windows of three electrolytes and recorded the cyclic voltammetry (CV) curves of three electrodes in NaClO_4_-based electrolytes using three-electrode cells. Notably, only a minor oxygen evolution reaction (OER) occurred during the charging process of 5 NaClO_4_ at −20 °C, reducing the likelihood of battery failure under low-temperature conditions (as shown in Figure 8).

At low temperatures, sodium salts participate in electrode interface reactions, ref. [30], affecting the composition and stability of the SEI membrane at the solid electrolyte interface. At the negative electrode, the reduction in hydrated Na^+^ in the electrolyte at a low concentration generates an ice-containing SEI membrane, which is loose in structure and poor in conductivity, while NaClO_4_ crystals precipitate in the electrolyte at high concentrations, leading to a local rupture of the SEI membrane, which triggers the continuous decomposition of the electrolyte. On the anode side, ClO_4_^−^ will be enriched on the cathode surface at high voltage, forming a high-impedance salt deposition layer, such as in the 17 m NaClO_4_ electrolyte, where the salt coverage on the cathode surface is more than 40% after charging at −30 °C, which hinders the de-embedding of Na^+^. This “selective interfacial blockage” mechanism of anode ice accumulation and cathode salt deposition is the core cause of low-temperature battery failure [57]. Among them, selective interfacial blocking refers to the phenomenon in battery or electrochemical systems where a specific material or interfacial layer can selectively hinder the passage of certain substances (such as solvent molecules, large-sized ions, or impurities) across the electrode/electrolyte interface, while allowing target ions (such as lithium ions and sodium ions) to migrate unimpeded.

### 2.3. Cathode and Anode Materials

#### 2.3.1. Structural Stability of Cathode Materials Is Affected at Low Temperatures

Current SIB cathode materials are mainly focused on polyanion materials (Na_3_V_2_(PO_4_)_3_, NVP), layered transitional metal oxide materials (TMOs), and Prussian blue analogs (PBAs). At low temperatures, the ion diffusion rate among the electrodes significantly slows down, and structural stability is also affected. The comparative table of polyanion materials, layered transition metal oxide materials, and Prussian blue analogs tested under low-temperature conditions is shown in Table 2.

Polyanionic materials occupy an important position in the research of low-temperature cathode materials due to their unique three-dimensional backbone structure. This structure consists of polyhedral anionic units, which endow the material with strong covalent bonding while constructing stable sodium ion diffusion channels, enabling the material to maintain a long cycle life even under high potential conditions. However, the large anionic groups are an obstacle to the improvement of their performance, limiting the electronic conductivity of the material itself and resulting in a low specific capacity, which makes it more difficult to achieve the desired energy density target at low temperatures [58,59,60]. NASICON-type polyanionic compounds are typical representatives of this, with the general formula Na_3_M_2_(XO_4_)_3_, where M covers elements such as V, Fe, Ni, Mn, Ti, etc., and X contains elements such as P, S, and Si. The three-dimensional open migration path of sodium ions possessed by such compounds gives them a unique advantage at low temperatures [28,61,62]. Na_3_V_2_(PO_4_)_3_, as a much talked about cathode material in the NASICON group of compounds, has a stable three-dimensional open framework consisting of a combination of angle-sharing PO_4_ tetrahedra and VO_6_ octahedra, which provides sufficient sodium ion migration space [63].

**Figure 9:**
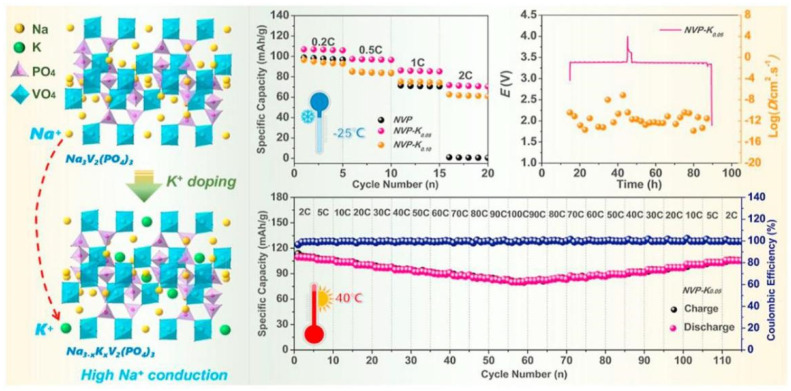
The schematic illustration of the structural stabilization of NVP electrode materials by doping K ions into the Na sites, along with the rate capability plots and cycling plots of the NVP sample. Copyright 2023 Elsevier [64].

Shen et al. [64] have achieved a K^+^-ion-doped NVP cathode via a rational approach, which increased the unit cell volume and widened the migration channels for Na^+^ ions. The schematic diagram of the specific structure is shown in Figure 9. When tested at a low temperature of −25 °C, the capacity of the undoped NVP electrode dropped sharply to zero at the current density of 2 C, while the capacity of NVP-K_0.05_ could still be maintained at around 72 mAh g^−1^. This mainly benefitted from the introduction of an appropriate amount of potassium (K) atoms that can not only keep the structural stability of NVP frameworks but also provide suitable migration channels for Na^+^ ions. The rate performance of the NVP-K_0.05_ cathode material is comparable to that of the state-of-the-art NVP in SIBs. Among all the Na_3-x_K_x_V_2_(PO_4_)_3_ electrode materials, the NVP-K_0.05_ electrode sample has the best Na migration kinetics, with a Na^+^ diffusion coefficient value that can reach 10^−11^ cm^2^ s^−1^, which is significantly superior to that of NVP.

TMO cathode materials can be divided into two categories based on the arrangement of sodium ions and transition metal layers: the P-type and the oxygen-type (O-type). In the P-type cathode structure, sodium ions occupy a prismatic position, while in the O-type cathode, sodium ions are in an octahedral position. Currently, the chemically stable P2-type and O3-type layered materials have attracted much attention, which have the stacking characteristics of ABBA and ABCABC, respectively, and occupy an important position in the research field by virtue of their excellent electrochemical properties [65]. However, these layered materials suffer from significant drawbacks. In the highly depolarized state, they undergo irreversible structural phase transitions, such as P2-OP4 or P2-O2 phase transitions. At the same time, intracrystalline cracks and phase transitions can cause volume changes of up to 23% in the material, which in turn leads to the collapse of the layered structure and severely affects the capacity retention and cycling performance of the material [66,67]. Researchers have actively explored improvement strategies. Liu et al. [68] synthesized a P2-type Na_0.696_Ni_0.329_Mn_0.671_O_2_ material with highly superior Na^+^ ion occupied locations, which achieved stable cycling at low temperatures down to −30 °C. The material is also suitable for the production of electrodes in the solid phase. In addition, adjusting the morphology and structure of the cathode material and reducing the grain size to the nanoscale can effectively shorten the diffusion distance of sodium ions in the solid phase and enhance the electrochemical reaction kinetics. Hwang et al. [69] proposed an innovative method to prepare high-density spherical particles consisting of radially assembled columnar structures. The chemical composition of the particles showed a gradient, with a high nickel (Ni) content NaNi_0.75_Co_0.02_Mn_0.23_O_2_ in the inner core and a Ni_0.58_Co_0.06_Mn_0.36_O_2_ shell with low Ni and Mn concentrations. This unique structure promotes the redox reaction of Ni^2+/3+/4+^ to take place mainly inside the material, which reduces the contact resistance at the cathode/electrolyte interface and effectively inhibits unnecessary side reactions, and demonstrates excellent cycling stability at low temperatures of −20 °C.

Prussian blue analogues (PBAs) have been widely studied in the field of cathode materials for SIBs by virtue of the significant advantages of an easy preparation process and possession of high capacity. However, all three common cathode materials have obvious performance shortcomings, and the presence of a large number of crystal defects and interstitial water limits their performance in low-temperature environments [70,71,72]. To solve this problem, researchers have explored optimized strategies. Zhang et al. [73] successfully prepared low-defect and sodium-rich PBA materials by using chelator- and sodium-assisted co-precipitation crystallization. Their synthesized Co_0.7_Ni_0.3_-hydrofluoric acid can achieve a capacity of 109 mAh g^−1^ at −30 °C without an activation process, which provides a new research direction for the performance enhancement of PBAs in low-temperature applications.

#### 2.3.2. The Lattice Instability of Anode Materials at Low Temperatures

Under the low-temperature environment, the anode materials of SIBs face the problems of sodium precipitation and unstable interfacial film. According to the sodium storage mechanism, the current anode materials are mainly divided into three categories, including intercalation type, alloying type, and conversion type.

**Figure 10:**
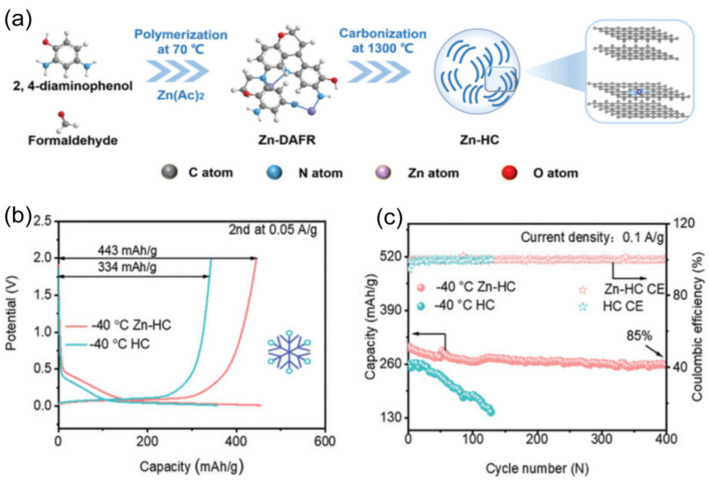
(**a**) Schematic illustration of the synthesis of Zn-HC. (**b**) The galvanostatic charge/discharge (GCD) curves (the second cycle). (**c**) Cycling stabilities. Copyright 2023 Wiley [73].

Non-graphitized hard carbon (HC) has become a typical material for carbon-based sodium storage anodes by virtue of its moderate layer spacing and abundant sodium storage sites [74,75]. Its porous structure endows the material with a large active surface area, but it also leads to an overconsumption of the active sodium source during the first charging to form the SEI film, making the initial efficiency low [30]. To address this issue, Hou et al. [76] prepared paper-like HC with an ordered fibrous structure using a pre-oxidation process, which improved the initial Coulombic efficiency of the material to 91.2%. The material showed excellent low-temperature cycling performance by maintaining a capacity retention rate higher than 80% after 1000 cycles at −15 °C and 500 mA g^−1^. In addition, the surface carbon coating technology can effectively inhibit the surface side reaction between HC and electrolyte and improve the initial efficiency, enabling conventional HC to achieve a high reversible capacity of 265 mAh g^−1^ at −15 °C and 0.1 C. The surface carbon coating technology can also effectively inhibit the surface side reaction between HC and electrolytes and improve the initial efficiency. However, HC materials suffer from the shortcoming of poor multiplication performance at low temperatures. It was found by Ponrouch et al. [77] that the introduction of heteroatoms such as nitrogen, phosphorus, sulfur, and oxygen can significantly improve the multiplicity performance and specific capacity by modulating the defects and layer spacing. A zinc single atom-regulated hard-carbon (Zn-HC) composite developed by Lu’s team [78], which combines monoatomic zinc with HC, possesses extended layer spacing (d_(002)_ = 0.408 nm), a highly developed nanopore structure, and fewer defects. These properties not only guarantee a high initial Coulombic efficiency but also accelerate the sodium ion storage process. Of particular interest is that the Zn-N_4_-C structure in the material can act as a catalyst to promote the rapid decomposition of NaPF_6_ and the formation of a thin and inorganic-rich SEI film, which further enhances the kinetics of sodium ion storage at the interface. After comprehensive electrochemical optimization, the Zn-HC material can maintain a capacity of 140 mAh g^−1^ at a high charging current of 0.05 A g^−1^, and its specific capacity is still as high as 443 mAh g^−1^ even at the extreme low temperature of −40 °C. After 400 cycles, the specific capacity of the Zn-HC material can be maintained at a high charge current of 0.05 A g^−1^, and the specific capacity is still as high as 443 mAh g^−1^ after 400 cycles, and the capacity retention rate is up to 85% after 400 cycles (as shown in Figure 10).

Zinc has a high crustal abundance (approximately 78 ppm), and its price is significantly lower than scarce metals like lithium and cobalt (the current zinc price is about USD 2.5/kg, less than 1/20th of the price of lithium carbonate). Additionally, the amount of zinc used in the doping process is typically low (atomic ratio of 5–15%), controlling material costs from the source. In cost-sensitive large-scale energy storage fields (such as grid peak regulation and low-speed electric vehicles), the zinc-doped hard carbon’s balance between capacity retention (>85% after 1000 cycles) and cost gives it greater promotion potential compared to high-priced silicon-based anodes. However, in high-end 3C products, attention must be paid to the long-term impact of zinc residues on interfacial stability to avoid hidden costs due to cyclic degradation. In summary, the cost competitiveness of zinc-doped hard carbon stems from elemental abundance and process adaptability, but its practical application requires optimization based on the performance-cost threshold of specific scenarios.

The research on alloying-type anode materials mainly focuses on the elements of germanium (Ge), stannum (Sn), and plumbum (Pb) belonging to the IVA group of the periodic table, and P, antimony (Sb), and bismuth (Bi) in the VA group, which can be prepared by the chemical reaction of forming sodium alloy phases, such as NaGe, Na_15_Sn_4_, Na_15_Pb_4_, Na_3_P, Na_3_Sb, Na_3_Bi, and so on [79,80]. In comparison to the insertion-type negative electrode materials, the alloy anode has many significant advantages: its theoretical specific capacity is more than 400 mAh g^−1^, the working potential is in the suitable interval of 0.2–0.6 V, and not only is the conductivity good, but also the raw material source is widely available. Based on these characteristics, alloy anodes have great potential for development in the field of anode materials for low-temperature SIBs [81,82,83]. In practical research and application, many research teams have achieved a series of results. Huang et al. [84] prepared layer-stacked Sb@graphene (LS-Sb@G) micro- and nano-composites using the solvent-thermal method, in which Sb nanoparticles were uniformly adhered to the graphene substrate and embedded in the intercalation to form a dense layered micro- and nanostructure. The capacity retention rate of this material was maintained at 63.7% after 100 cycles at −20 °C. Chen et al. [85] successfully prepared Sb composite-coated three-dimensional porous carbon microspheres by applying a salt-template-guided spray-drying strategy, in which the N/S-co-doped porous shell structure was able to effectively confine antimony nanocrystals, which enables the material to maintain excellent high-fold cycling performance even at a low temperature of 5 °C.

Currently, research on negative electrode materials for conversion reactions focuses on transition metal sulfides (TMSs), phosphides, and selenides, which have attracted much attention for research in low-temperature environments by virtue of their higher electronic conductivity and faster reaction kinetic [86,87,88]. Unlike conventional carbon-based materials, these anode materials for conversion reactions rely on a unique reversible redox reaction to achieve sodium ion storage, which provides the advantage of higher theoretical capacity and energy density. However, they suffer from a similar problem to alloy anodes in that they undergo significant volume changes during cycling, leading to material fracture and disruption of the electrode contact, which in turn results in rapid capacity degradation [89,90]. Researchers have carried out in-depth studies to address the above problems and achieved results. Jiang et al. [91] took MoSe_2_ as the object of study, and after analyzing its basic chemical reaction process, it was found that the reversibility of the conversion reaction depended on whether Mo, the discharge product of MoSe_2_, could react with Na_2_Se to achieve the regeneration of MoSe_2_. Based on this, the reversible reaction is promoted by modulating the structural strain of the Mo_2_ material to change the activities of the transformation products molybdenum (Mo) and Na_2_Se. Among them, the TS-MoSe_2_ material with tensile confinement exhibits highly reversible sodium ion storage performance even at the extreme low temperature of −30 °C. After 100 cycles at a current density of 0.1 A g^−1^, the reversible capacity of TS-MoSe_2_ was still as high as 380 mAh g^−1^. Fan et al. [92] used a solvent-thermal method to design micro- and nanostructured FeS spheres (FeS@ g-C) coated with thin graphitic carbon (g-C), which is similar to the design idea of the alloying reaction of carbon composites; g-C coating enhances the temperature resistance of porous gradient FeS sphere composites by virtue of short ionic diffusion paths and high conductivity. The FeS@ g-C composites reach a reversible capacity of 311 mAh g^−1^ after 80 cycles at −25 °C and 0.05 A g^−1^.

**Figure 11:**
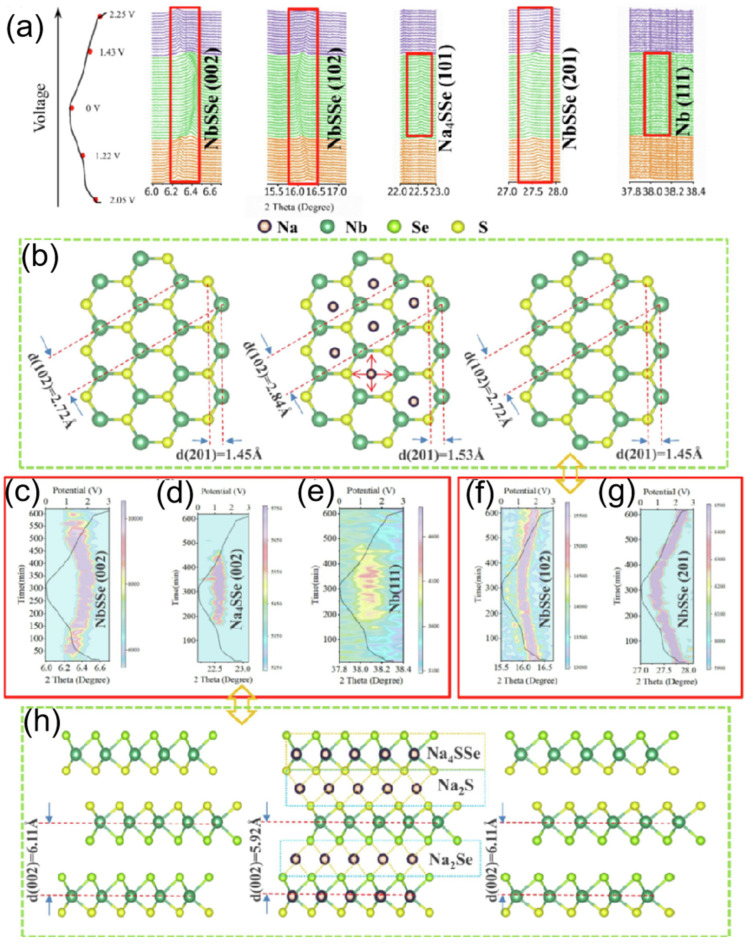
(**a**) Synchrotron X-ray powder diffraction was used, and superimposed voltage distribution plots are shown. (**b**) Schematic diagrams of changes in NbSSe. (**c**,**f**,**g**). The selected 2θ ranges for NbSSe. (**d**,**e**) The selected 2θ ranges for Na_4_SSe and Nb, respectively. (**h**) Schematic diagrams of changes in the NbSSe (002) lattice planes. Copyright 2022 Elsevier [93].

To deeply investigate the sodium ion intercalation behavior and conversion reactions, in situ synchrotron X-ray diffraction (XRD) was conducted to monitor the reaction process, as shown in Figure 11a. For the intercalation and deintercalation reactions, the process is continuous across the entire voltage range, with corresponding reactions occurring between the lattice layers of NbSSe. Taking the (102) and (201) crystal planes as examples (as shown in Figure 11b,f,g), the NbSSe structure gradually changes along with sodium ion intercalation/deintercalation throughout the charge/discharge process. After charging, according to Bragg’s law, the lattice spacing of the (102) plane increases from 2.72 Å to 2.84 Å. Taking the conversion mechanism as an example (illustrated in Figure 11c–e,h), when the voltages are <1.22 V (discharge) and >1.45 V (charge), the reaction kinetics are mainly governed by the synergistic driving of thermodynamics and kinetics. During the continuous charge/discharge process, when the voltage is between 1.22 V and 1.45 V, conversion reactions occur, accounting for <37% of the total capacity (as shown in Figure 11d,e). The formation energies of Na_2_S, Na_2_Se, and Na_2_SSe are −1.292 eV, −1.069 eV, and −1.176 eV, respectively. As schematically shown in Figure 11h, considering the influence and proximity of formation energies, the binding force of forming Na_2_S and Na_2_Se (due to interlayer sodium ions capturing partial S or Se atoms) causes the (002) lattice plane spacing to decrease [93,94].

### 2.4. Electrode/Electrolyte Interface

**Figure 12:**
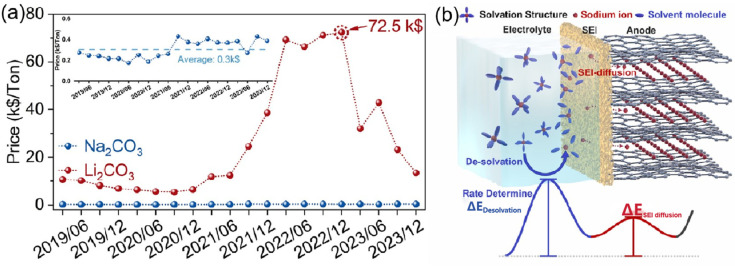
(**a**) Metric ton price trend of sodium carbonate and battery-grade lithium carbonate; the inset is the enlarged price trend of sodium carbonate. (**b**) Schematic illustration of sodium-ion behavior during charging in the sodium-ion full-cell and the energy changes (color online). Copyright 2024 Springer Nature Link [97].

In a low-temperature environment, the interfacial compatibility between the electrolyte and electrode materials decreases significantly, resulting in an increase in charge transfer resistance and an increase in the polarization phenomenon of the battery during charging and discharging, which seriously affects the charging and discharging efficiency and cycling stability and ultimately leads to a decrease in the capacity of the battery. The formation of an SEI film at the interface between the electrode and electrolyte plays a decisive role in battery performance. A stable SEI film can effectively prevent the continuous decomposition of the electrolyte, optimize the ion transport process, and inhibit the growth of dendrites, thus significantly improving the cycle stability and safety of the battery. The solvated structure and composition of the electrolyte are the key factors affecting the formation of SEI film. High-concentration electrolytes and localized high-concentration electrolytes are capable of forming anion-rich solvated structures, which contribute to the formation of inorganic-rich SEI films on the surface of sodium metal anodes. In addition, weakly solvated electrolytes and dilute weakly interacting electrolytes are also conducive to the generation of stable SEI films. Taking the low-temperature-adapted electrolyte as an example, the anion-rich solvated structure can promote the formation of a uniform and continuous SEI film on the surface of a hard-carbon anode, which significantly improves the low-temperature performance of the battery [95,96]. The diffusion process of Na^+^ ions at the interface (mainly the SEI film) consumes energy, and an excessively thick SEI film on the anode surface can severely limit the kinetic performance of the battery in low-temperature environments, as shown in Figure 12. Therefore, electrolytes that can form a thin interfacial layer are more advantageous in low-temperature applications and help to improve the overall performance of the battery [97].

**Figure 13:**
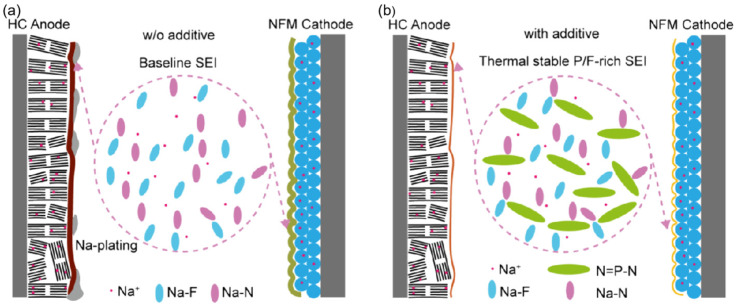
Schematic of the electrode SEI structure. (**a**) Without additives. (**b**) With additive. Copyright 2025 American Chemical Society [98].

Studies focus on using fluorinated additives to precisely tailor the composition of SEI films, thereby further improving the cycling stability and safety of SIBs, especially under low-temperature conditions. Zhang et al. [98] demonstrated that a Na_0.98_Ni_0.5_Fe_0.2_Mn_0.3_O_2_ (NFM) hard-carbon (HC) pouch cell with 10 wt.% ethoxy (pentafluoro) cyclotriphosphazene (PFPN) exhibits an 85% capacity retention after 450 cycles at a 0.2 C rate and 0 °C, showing about a 4% increase in capacity compared to the baseline electrolyte (81.33%). At 0.5 C and 30 °C, the capacity retention remains 84% after 600 cycles. These results indicate that the addition of PFPN leads to the formation of a unique SEI structure during long-term cycling, with the electrode surface enriched in P and N elements. This structure effectively enhances the battery’s kinetic performance and suppresses sodium precipitation, as shown in Figure 13. Thus, during electrochemical cycling, PFPN facilitates the formation of a distinct SEI layer rich in phosphorus and nitrogen, thereby improving the battery’s kinetic characteristics.

**Figure 14:**
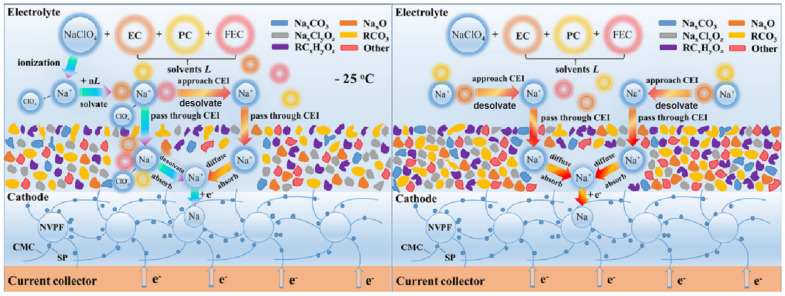
Different behavior of Na^+^ across the CEI layer. Copyright 2022, Elsevier [99].

During the operation of SIBs, the electrolyte reacts with the surface of the cathode to form a CEI film. The performance of CEI film has a crucial impact on the overall performance of the battery. A suitable electrolyte can induce the cathode surface to generate a thin and strong CEI layer, which is a protective film that can effectively prevent the electrode material from coming into direct contact with the electrolyte. This protective film can effectively avoid direct contact between the electrode material and the electrolyte, thus improving the cycle life of the battery at low temperature conditions [99]. The specific mechanism is shown in Figure 14.

## 3. Strategy

### 3.1. Strategies for Improving the Low-Temperature Performance of SIB Electrolytes

As the primary medium connecting the cathode and anode, the electrolyte dictates the Coulombic efficiency, energy density, and cycle life of the battery system. To ensure the normal operation and performance of batteries at low temperatures, the electrolyte needs to exhibit excellent electrical conductivity, superior fluidity, and strong polarization resistance. These characteristics are of significance for enhancing the rate capability and fast-charging performance of SIBs under extremely cold conditions. The main strategies for optimizing the electrolyte include the selection of sodium salts, the addition of additives, and the utilization of multiple solvents to form a more favorable SEI [97]. These strategies optimize the electrochemical stability and ionic conductivity of the electrolyte, reduce its viscosity, and enhance the ion mobility and diffusion coefficient, thereby maintaining high power output and cycling stability at low temperatures. However, the selection of appropriate sodium salts requires a delicate balance among their solubility, ionic conductivity, and chemical stability to ensure the optimal performance of the electrolyte. Although the addition of additives is beneficial for adjusting the properties of the electrolyte, it increases complexity and necessitates careful management of their types and concentrations to prevent undesirable side reactions. Additionally, while the adoption of multi-solvent systems enhances the capabilities of the electrolyte, it may increase the weight and cost of the battery system. A thorough assessment of compatibility between the various solvents is also required to reduce the risk of phase separation, which could compromise the overall efficiency and reliability of the battery. Nevertheless, the significance of the solvation structure within the electrolyte must not be overlooked [100].

#### 3.1.1. Solvent Component Regulation

**Figure 15:**
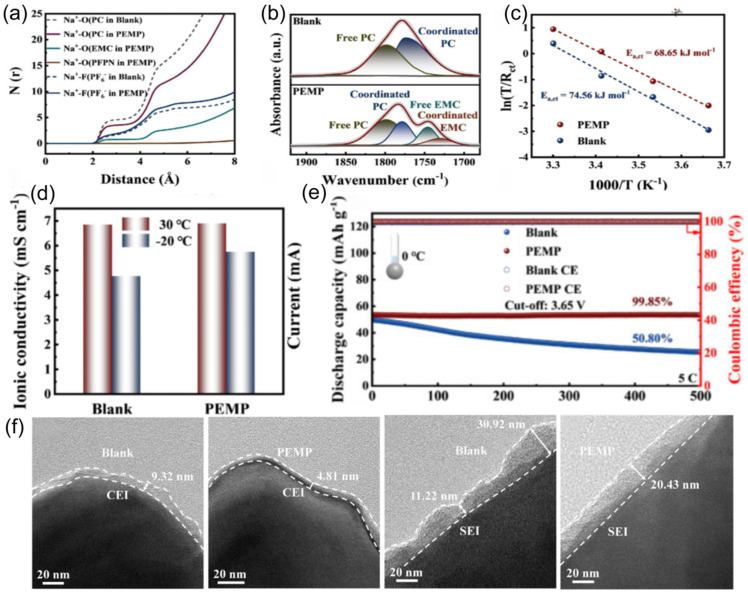
(**a**,**b**) The coordination number and FTIR spectra of different electrolytes. (**c**) The E_a,ct_ analyzed by Arrhenius. (**d**) Ionic conductivity of different electrolytes. (**e**) GCD curve of the NFP||Na battery at 5C at 0 °C. (**f**) HR-TEM images of the NFP cathode and HC anode surfaces of the NFP||HC battery surface after cycling in blank and PEMP electrolytes. Copyright 2024 Elsevier [53].

The coordination ability of solvent molecules with cations fundamentally determines the desolvation process. This means that the ability of solvent molecules to effectively interact and coordinate with cations determines the efficiency with which these cations are stripped of their solvent shell layers. Strong coordination may lead to co-intercalation of cation/solvent complexes, inevitably damaging the structure of electrode materials and exacerbating side reactions in the solid phase. In the study conducted by Wang et al. [101], the co-solvent 1,3-dioxolane (DOL) optimizes the solvated structure of the electrolyte due to its low melting point and low solvation energy. This co-solvent enhances the participation of more PF_6−_ anions in the formation of contact-ion pairs (CIPs) and reduces the desolvation energy of Na^+^. The electrolyte remains in a stable liquid state with good ionic conductivity at −40 °C, which facilitates the formation of a stable NaF-rich SEI membrane and improves the low-temperature performance of SIBs. At the same time, extremely weak interactions between cations and solvents impede ion migration during the charging and discharging processes. Therefore, mixing multiple solvents and adjusting their proportions can optimize the solvation structure, thereby achieving optimal electrochemical performance. In low-temperature and high-rate scenarios, the properties of SIBs are limited by the kinetic and thermodynamic properties of sodium ions. Therefore, the precise regulation of electrolyte solvation structures has emerged as a pivotal approach to overcoming performance bottlenecks. Li et al. [53] innovatively proposed a “hierarchical doping” strategy [102], using PC as the main solvent and introducing EMC and 1,1,2,2-tetrafluoroethyl-2,2,3,3-tetrafluoropropyl ether (PFPN) as an additive. EMC can penetrate into the first solvation layer of sodium ions (Figure 15a,b), reducing the desolvation energy barrier and dispersing the electrolyte. PFPN resides in the second solvation layer, weakening the interaction between ions and solvents through a “tug-of-war effect” and participating in the formation of a stable interfacial film. The designed Na button battery containing the PC, EMC, and PFPN mixed solvent electrolyte (PEMP) electrolyte rebuilds the solvation structure of sodium ions, enhancing ionic conductivity (Figure 15c) and the diffusion rate within the electrolyte (Figure 15d). Notably, the Na_3_Fe_2_(PO_4_)P_2_O_7_ (NFP)||Na coin cell with this electrolyte exhibits outstanding performance at −20 °C, maintaining a capacity retention rate of over 99% after 500 cycles at 5C (Figure 15e). After cycling the same number of times in the blank electrolyte and the PEMP electrolyte, the CEI and SEI formed in the PEMP electrolyte are thinner and more uniform (Figure 15f). This design, for the first time from the perspective of hierarchical regulation of solvation structures, demonstrates the feasibility of multi-component solvent synergistic optimization in enhancing the transport efficiency of sodium ions.

**Figure 16:**
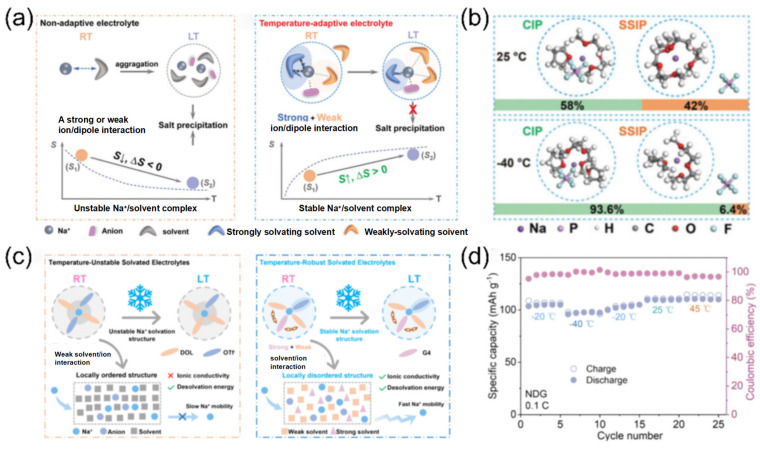
(**a**) Schematic illustration showing the low-temperature electrolyte design strategy. (**b**) The representative solvation structures; the bottom shows the percentage of each solvation configuration. Copyright 2023 Elsevier [103]. (**c**) Schematic illustration of temperature-unstable solvation structures and temperature-robust solvation structures. (**d**) Charge/discharge capacities at various temperatures. Copyright 2025 American Chemical Society [104].

Following the concept of the dynamic regulation of solvation structures, researchers have discovered that the combination of strong and weak solvation properties in ether-based mixed solvents can endow electrolytes with temperature-adaptive characteristics. The Yang team [103] designed a temperature-adaptive electrolyte. Taking diglyme (DIG) and THF as examples, these solvents have similar dielectric constants and donor numbers. When mixed, the solvation structure can spontaneously transform at low temperatures, preventing salt precipitation (Figure 16a). As the temperature decreases, more THF participates in the solvation process (Figure 16b), stabilizing the sodium ion/solvent complexes and increasing the entropy of the system. Similarly, Huang et al. [99] designed an electrolyte with a temperature-robust solvation structure. The mixed solvent of tetraethylene glycol dimethyl ether (G4) and 1,3-dioxolane (DOL) forms a temperature-robust solvation structure through intermolecular forces anchoring (Figure 16c). The Na_3_V_2_(PO_4_)_3_||Na full battery demonstrates a reversible capacity of 95.9 mAh g^−1^ at −40 °C, which is 87.6% of its room-temperature capacity (Figure 16d). This marks a technological upgrade of solvation structures for electrolytes, maintaining robust temperature stability rather than volatility.

**Figure 17:**
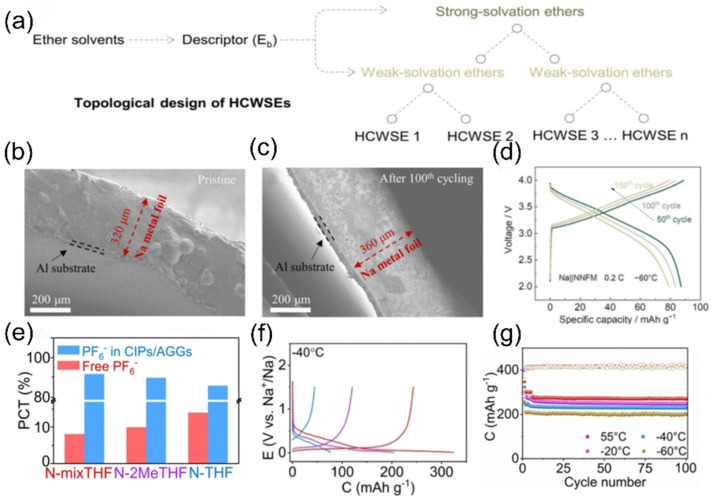
(**a**) Schematic diagram of the topological design. (**b**,**c**) Cross-sectional SEM images of a Na metal anode and the one after 100 cycles. (**d**) Voltage profiles of the Na||NNFM cell at −60 °C. Copyright 2025 American Chemical Society [106]. (**e**) Coordinated structures. (**f**) Charge/discharge curves of HC at 50 mA g^−1^ at −40 °C. From right to left, N-mixTHF, N-2MeTHF, and NTHF. (**g**) Cycling performance at different temperatures. Copyright 2024 Wiley [107].

For the extreme condition of ultra-low temperature (below −60 °C), two complementary strategies have further expanded the boundary of solvation structure regulation. First, the Wang team [105] introduced strong solvation ether additives into the weak solvation system through topological design (Figure 17a) [106]. Cross-sectional SEM images of the pristine Na metal anode (Figure 17b) and after 100 cycles (Figure 17c) illustrate structural stability. The voltage profiles of the Na||NNFM cell at 0.2 C at −60 °C are shown in (Figure 17d). Second, Fang et al. [107] introduced 2-methyltetrahydrofuran (2MeTHF), which has weak solvating ability, into THF to construct a weakly solvating electrolyte. This weakens the Na^+^-solvent/dipole interaction, prompting more anions to participate in the solvation sheath (Figure 17e) and form a low-resistance SEI film with rich NaF. As a result, hard carbon electrodes achieve reversible capacities of 243.2 mAh g^−1^ and 205.4 mAh g^−1^ at −40 °C and −60 °C (Figure 17f,g). Although these two approaches differ in methodology, they jointly verify the universal principle of optimizing low-temperature mass transfer and interfaces by regulating the strength of ion/solvent interactions.

With the deepening understanding of solvation structure regulation, researchers have turned their attention to a broader range of battery systems. Although LIBs differ from SIBs in ionic properties, there are similarities in the underlying logic of electrolyte optimization. The Mo team [108] applied a moderately fluorinated ethyl difluoroacetate (EDFA) solvent to balance weak binding energy and high ionic conductivity, promoting the formation of a low-resistance inorganic SEI film on the graphite anode. This enabled a reversible capacity of 279 mAh g^−1^ and 6 C fast charging at −40 °C. Gao et al. [109] utilized a synergistic system of methyl propionate and methyl pentafluoropropionate. By adjusting the Li^+^ solvation sheath through hydrogen bonding, they weakened the Li^+^-solvent interaction and facilitated rapid desolvation at the interface. As a result, the LiFePO_4_ (LFP)||graphite battery maintained 81.2% of its room-temperature capacity at −30 °C, and a 1 Ah pouch battery still had a discharge capacity of 0.85 Ah at −20 °C, demonstrating the potential of synergistic optimization of non-fluorinated and fluorinated solvents.

The cutting-edge explorations in solvation structure design and interfacial chemistry regulation of low-temperature LIBs have provided valuable technical inspirations for low-temperature SIBs. Strategies such as the synergistic regulation of ion/solvent interactions through strong/weak solvents [110] and the promotion of low-resistance inorganic interfacial film formation via fluorination modification essentially revolve around the common goal of “reducing the desolvation energy barrier of ions and enhancing bulk phase transport and interfacial stability”. SIBs can draw on these molecular-level design concepts. By regulating the dielectric constant, donor number, and degree of fluorination of solvents, the solvation structure of Na^+^ can be optimized, and the formation of a stable interfacial layer rich in NaF can be promoted. This approach enables the enhancement of ionic conductivity and interfacial compatibility at low temperatures at the same time, offering a cross-system technical pathway to overcome the kinetic limitations of sodium ions.

In the recent past, aqueous low-temperature solid-state SIBs have gained some traction. Gui et al. [111] have built a bendable quasi-solid aqueous low-temperature solid-state SIB with the help of a glycol–polyacrylamide–sodium perchlorate hydrogel electrolyte. This innovative design inhibits icing through hydrogen bonding between glycol and water molecules, enabling the battery to maintain 64% of its room-temperature energy density and achieve an ionic conductivity up to 2.5 mS cm^−1^ at −30 °C. Additionally, the battery exhibits excellent mechanical toughness, allowing it to be bent while reliably supplying power to electronic devices.

In the future, based on the molecular design of polymer electrolytes and the synergistic optimization strategy of composite interfacial layers, we anticipate the development of a high-performance SIB system adapted to a wide temperature range. This research focuses on the design of polymer chain segment polarity, the construction of ion conduction networks within the interfacial layer, multicomponent synergistic interfacial engineering, and the development of novel composite electrolytes. It systematically investigates multidimensional approaches to optimize the interfacial dynamics of the batteries and enhance ion transport in low-temperature environments. By regulating the microscopic interactions of the electrolyte and the multiscale structure of the interface, this research provides a solid theoretical foundation and practical framework for overcoming the core challenges of interfacial impedance accumulation and ion migration hysteresis under extreme operating conditions. Furthermore, it facilitates the extensive application of SIBs in scenarios requiring high cold resistance, high power, and flexibility.

#### 3.1.2. Efficient Dissociation of Sodium Salts

In SIBs, the optimization of electrolyte formulations is highly dependent on the properties of the selection of sodium salts. Sodium salts such as sodium hexafluorophosphate (NaPF_6_), sodium tetrafluoroborate (NaBF_4_), sodium perchlorate (NaClO_4_), sodium bis(trifluoromethanesulfonyl)imide (NaTFSI), sodium fluoro–bis(trifluoromethanesulfonyl)imide (NaFTFSI), and sodium fluorosulfonylimide (NaFSI) play a crucial and multi-dimensional role in the electrochemical performance of the batteries. The development of novel sodium salts with appropriate anion structures and properties can improve the solvation state of sodium ions and facilitate desolvation. For example, the design and synthesis of sodium salts with larger anion volumes and more stable structures can make the solvation shell around sodium ions looser and facilitate desolvation. Additionally, optimizing the concentration of sodium salts also impacts the desolvation process. Appropriately increasing the salt concentration can increase the number of ion pairs in the electrolyte, altering the solvation structure and promoting desolvation to a certain extent. However, excessively high salt concentrations may lead to increased electrolyte viscosity and hindered ion migration, necessitating the identification of an optimal concentration balance. In the work of Guo et al. [112], the localized high concentration electrolyte (LHCE) facilitates the exchange of anion/solvent molecules within the solvated sheaths of sodium ions and organic cations through the introduction of an ionic liquid. This process reduces the size of the solvated clusters and enhances sodium/anion coordination, which in turn promotes rapid ionic dynamics both in the liquid phase and at the interface. As a result, this enables longer-lasting low-temperature sodium deposition and contributes to the development of a shuttle-effect-free 0.5 Ah sodium–sulfur flexible-pack battery. As the core conductive substances, the Na^+^ ions dissociated from these sodium salts upon dissolution serve as the primary charge carriers that shuttle between the cathode and anode electrodes during charging and discharging. The regulation of their concentration directly affects the ionic conductivity of the electrolyte. By maintaining ion balance between the cathode and anode electrodes, polarization can be effectively suppressed, internal resistance can be reduced, and rapid ion migration can be promoted. Sodium salts are also deeply involved in the formation of the SEI. The anions dissociated from sodium salts collaborate with solvent molecules to construct an ion-selective SEI film, which not only blocks electron transfer to inhibit side reactions but also provides an efficient transport pathway for Na^+^ ions, directly determining the interface stability and the cycle life of the battery. Moreover, the electrochemical window of the sodium salt anions needs to encompass the redox potential ranges of both the cathode and anode electrodes to ensure the battery’s thermodynamic stability. In terms of thermal stability, common sodium salts exhibit a decreasing trend as follows: NaClO_4_ > NaBF_4_ > NaTFSI > NaPF_6_ > NaFTFSI > NaFSI [113]. Although NaClO_4_ demonstrates excellent thermal stability due to the high oxidation state of chlorine, its strong oxidizing property makes it prone to triggering violent oxidation reactions, thus limiting its practical application. Conversely, while NaPF_6_ has relatively lower thermal stability, it has become the current mainstream choice due to its lowest oxidation potential and highest electrical conductivity. These property disparities underscore that the formulation design of electrolytes for SIBs requires a precise balance among ionic conduction efficiency, interface stability, and thermal safety to meet the high-performance requirements of diverse occasions [114].

**Figure 18:**
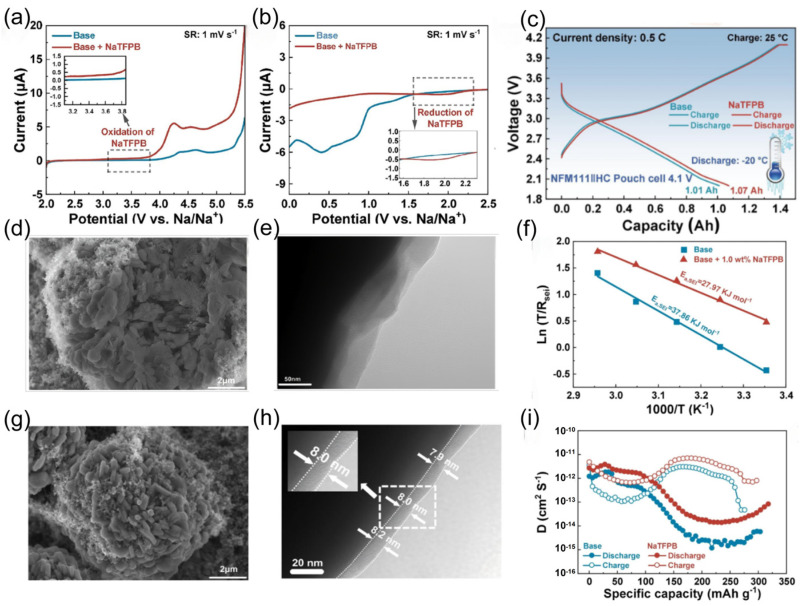
(**a**,**b**) LSV spectra of base and base+NaTFPB electrolytes at 1 mV s^−1^. (**c**) The discharge comparison at a low temperature of −20 °C. (**d**,**e**,**g**,**h**) SEM and TEM images of the NFM111 cathode after cycling in a base electrolyte and a NaTFPB-containing electrolyte. (**f**) The activation energies for the Na^+^ transport through the SEI. (**i**) GITT curves and diffusion coefficients of base + NaTFPB electrolytes. Copyright 2025 Elsevier [115].

Hu et al. [115] added the sodium salt sodium tetrakis(pentafluorophenyl)borate (NaTFPB) to the basic electrolyte. Through theoretical simulations and experimental studies, they found that NaTFPB can regulate the structure of the first solvation shell of Na^+^ [116], and preferential redox reactions (Figure 18a,b) occurred on the electrode surface, forming stable CEI and SEI layers rich in NaF and boron compounds. This effectively inhibits the decomposition of the electrolyte and the dissolution of transition metals, improves ion transport kinetics, and provides the battery with good discharge capability at −20 °C (Figure 18c). As shown in the SEM and Transmission Electron Microscope (TEM) images of the NaNi_0.33_Fe_0.33_Mn_0.33_O_2_ (NFM111) cathode after cycling in different electrolytes (Figure 18d,e,g,h), these layers effectively inhibited the decomposition of the electrolyte and the dissolution of transition metals. Analysis of the activation energy of Na^+^ transport through the SEI (Figure 18f) revealed that the addition of NaTFPB reduced the activation energy and facilitated ion transport. In addition, the Galvanostatic Intermittent Titration Technique (GITT) curves and diffusion coefficients of the alkali + NaTFPB electrolyte (Figure 18i) further indicated that the ion diffusion performance was improved.

**Figure 19:**
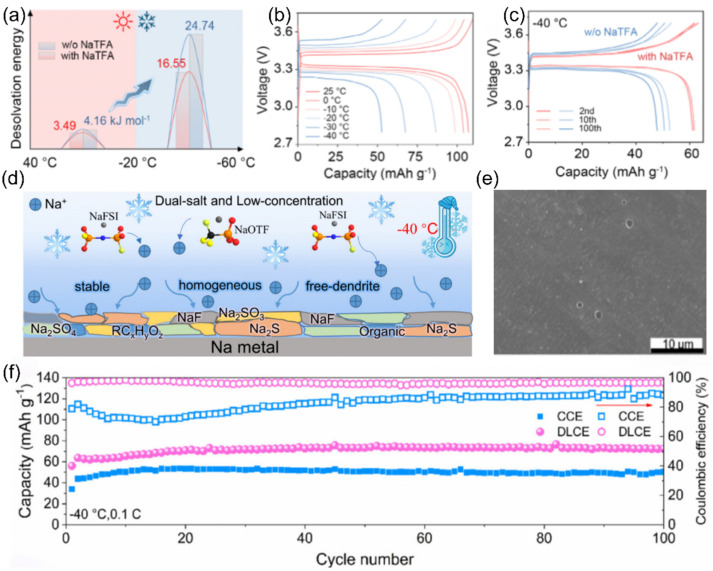
(**a**) Schematic of desolvation energy at a wide temperature. (**b**) Charge/discharge curves in the electrolyte with NaTFA. (**c**) Charge/discharge curves at 100 mA g^−1^ at −40 °C. Copyright 2024 PANS [117]. (**d**) Schematic diagram of the synthesis process. (**e**) SEM images of electrolytes after cycling. (**f**) Cycling performance of the Na||Na_3_V_2_(PO_4_)_3_ full-cells. Copyright 2025 American Chemical Society [118].

To address the severe capacity loss in SIBs caused by slow interfacial dynamics at low temperatures, the Li’s team [117] introduced the trifluoroacetate (TFA^−^) anion with ultra-high electron-donating ability into a diglyme (G2)-based electrolyte to regulate the solvation structure of sodium ions. Experimental results and theoretical calculations demonstrate that TFA^−^ can enhance its coordination with sodium ions, enabling more hexafluorophosphate anion (PF_6_^−^) anions and fewer G2 molecules to occupy the inner solvation sheath, thereby reducing the desolvation energy (Figure 19a). As a result, the Na||Na_3_V_2_(PO_4_)_3_ battery can retain 60.2% of its room-temperature capacity at −40 °C (Figure 19b) and 99.2% capacity retention after 100 cycles (Figure 19c).

Yang et al. [118] innovatively dissolved two sodium salts, sodium trifluoromethanesulfonate (NaOTf) and sodium hexafluorophosphate (NaPF_6_), in diglyme (G2) (Figure 19d) to prepare a weakly solvated electrolyte [119] with a small amount of additive. In the low-temperature environment of −40 °C, owing to the weak Na^+^-G2 interaction in the weakly solvated electrolyte, the surface of sodium metal remains smooth during cycling (Figure 19e) without dendrite formation. This good surface condition ensures the electrochemical performance of the battery. The half-cell has a high Coulombic efficiency of 99.2% after 100 cycles at −40 °C and 0.1 C, while being able to maintain a capacity of 70.5 mAh g^−1^ (Figure 19f).

**Figure 20:**
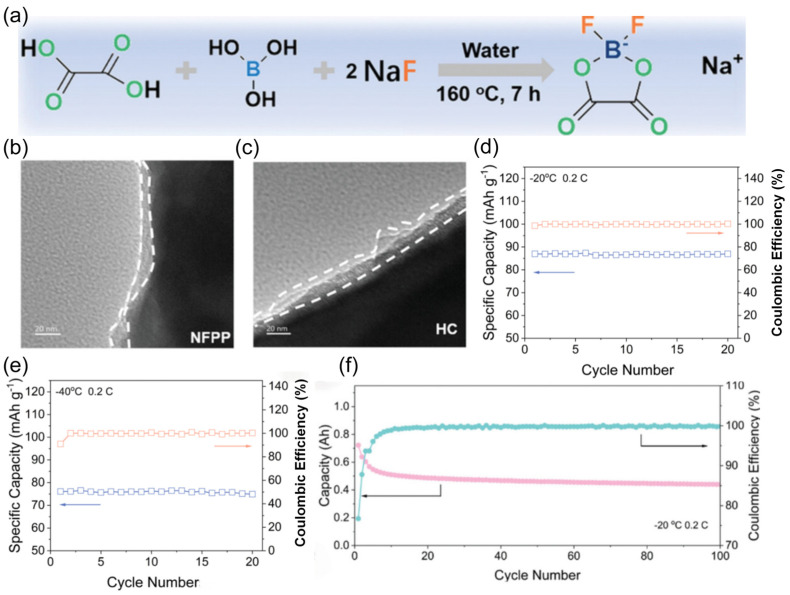
(**a**) A schematic illustration of the synthesis of NaDFOB. (**b**,**c**) TEM images of NFPP and HC electrodes after cycling. (**d**,**e**) Cycling performance of Na//NFPP half-cells at −20 °C and −40 °C. (**f**) Cycling performance of the HC//NFPP pouch at low temperature. An arrow to the left indicates that the curve corresponds to the left vertical axis, and the same is true to the right. Copyright 2024 Wiley [120].

Aiming at the shortcomings of commonly used sodium salts, the Xia team [120] developed a low-cost and easy-to-prepare sodium salt: sodium difluoro(oxalato)borate (NaDFOB) (Figure 20a). The synergistic effect of the oxalic acid root and fluorine atoms in its molecular structure forms a dense composite film of NaF/Na_2_B_4_O_7_ on the electrode surface (Figure 20b,c). The Na_4_Fe_3_(PO_4_)_2_P_2_O_7_ (NFPP) electrode exhibits excellent performance in the electrolyte containing NaDFOB, with a capacity retention rate of 92.7% at −20 °C and 84.8% at −40 °C (Figure 20d,e). For the HC//NFPP pouch full battery at −20 °C, with an initial capacity of 0.8 Ah, the capacity tends to stabilize after a certain number of cycles, and the final capacity is approximately 0.4 Ah, with the final Coulombic efficiency approaching 100% (Figure 20f), demonstrating the potential of the novel sodium salt in practical devices.

Furthermore, although a 2 m dual-salt sulfolane (TMS)/ethyl acetate (EA)-based local high-concentration electrolyte (LHCE) [121] has been designed, its outstanding low-temperature performance offers novel insights for further electrolyte design. In the future, through the synergistic effects of solvents and salts, it may be possible to develop similar low-temperature sodium salt materials. These studies, ranging from the screening of anion types to the regulation of solvation shell structures, interfacial film engineering, and the synthesis of novel sodium salts, systematically reveal the performance optimization pathways for low-temperature SIBs. They provide extensive theoretical and experimental support for overcoming the bottlenecks of energy storage technologies in extreme environments.

#### 3.1.3. Additives

In the electrolytes of low-temperature SIBs, additives form a multidimensional regulation system through functional differentiation and synergistic effects. In terms of composition, they can be classified into four major categories: solvent type, film-forming type, conductivity-enhancing type, and stability type. These categories, respectively, undertake core functions of lowering the freezing point, inducing the formation of interfacial films, optimizing ion transport, and suppressing side reactions.

The underlying mechanisms of their actions essentially involve reconstructing the Na^+^ solvation sheath [122] through coordination chemistry, improving the low-temperature viscosity of the electrolyte via the eutectic effect [123], and directionally constructing nanoscale dense interfacial films on the surfaces of cathode and anode electrodes through redox reactions. Meanwhile, with the help of chemical adsorption of functional groups containing phosphorus, sulfur, or cyano groups, or filling with nanoparticles, the mechanical properties of the interface are enhanced, and the growth of sodium dendrites and hydrofluoric acid (HF) corrosion are inhibited [124,125,126,127,128].

These mechanisms work in concert to enable the ionic conductivity of the electrolyte to reach the ideal value at low temperatures and promote a capacity retention rate of the battery in extreme low-temperature environments exceeding 90%, thus becoming a key technical factor in breaking through the low-temperature performance bottleneck of SIBs.

**Figure 21:**
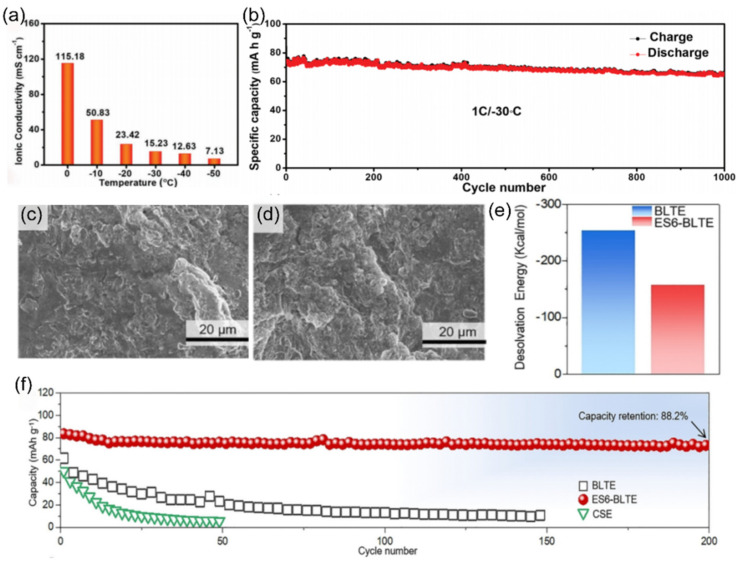
(**a**) The ionic conductivity of CaCl_2_ + NaClO_4_ electrolyte at different temperatures. (**b**) Cyclic performances of the full-cell under −30 °C. Copyright 2023 Wiley [121] (**c**,**d**) SEM images of Na anodes with BLTE and ES6-BLTE electrolytes after cycling. (**e**) The desolvation energy of BLTE and ES6-BLTE electrolytes obtained from DFT calculations. (**f**) Cyclability of the Na||NVP cells with BLTE and ES6-BLTE electrolytes at 0.1 C. Copyright 2023 WILEY [130].

When traditional aqueous electrolytes encountered the challenge of ion conduction failure at −50 °C, Zhu et al. [129] prepared a new low-temperature electrolyte by adding calcium chloride (CaCl_2_) as an antifreeze additive to a 1m NaClO_4_ aqueous electrolyte. At −50 °C, ionic conductivity reached a remarkable 7.13 mS cm^−1^ (Figure 21a), far exceeding the 0.11 mS cm^−1^ of the NaClO_4_ electrolyte with dimethyl sulfoxide (DMSO) addition. This breakthrough enabled the Na_2_CoFe(CN)_6_-activated carbon full-cell to exhibit outstanding performance at low temperatures. At −30 °C, the specific discharge capacity of the cell reached 74.5 mAh g^−1^, and after 1000 cycles, the discharge capacity remained at 64.6 mAh g^−1^, with a capacity retention rate of 86.7% (Figure 21b).

Zhong’s team [130] identified the kinetic bottleneck in the sodium ion desolvation process and aimed to enhance the low-temperature performance of SIBs by regulating the solvation structure with additives. They designed a carbonate-based electrolyte, 1.0 M NaFSI in EC/PC/DEC (1/1/4, *v*/*v*/*v*), containing 6% ethylene sulfate (ES6BLTE). As the core additive, ES optimized the solvation structure through molecular engineering, reducing the coordination between Na^+^ and solvent molecules. This led to a significant decrease in the Na^+^ desolvation energy from −253.1 kcal mol^−1^ in the baseline low-temperature electrolyte of 1.0 M NaFSI in EC/PC/DEC (1/1/4, *v*/*v*/*v*) (BLTE) electrolyte to −157.5 kcal mol^−1^ (Figure 21e), remarkably reducing the interfacial reaction resistance. This microscopic coordination reconstruction brought about a macroscopic performance leap. SEM images of Na anodes with BLTE (Figure 21c) and ES6BLTE electrolytes (Figure 21d) after cycling reveal structural differences. The Na||Na_3_V_2_(PO_4_)_3_ full-cell achieved a capacity retention rate of 88.2% after 200 cycles at 0.1 C (Figure 21f).

**Figure 22:**
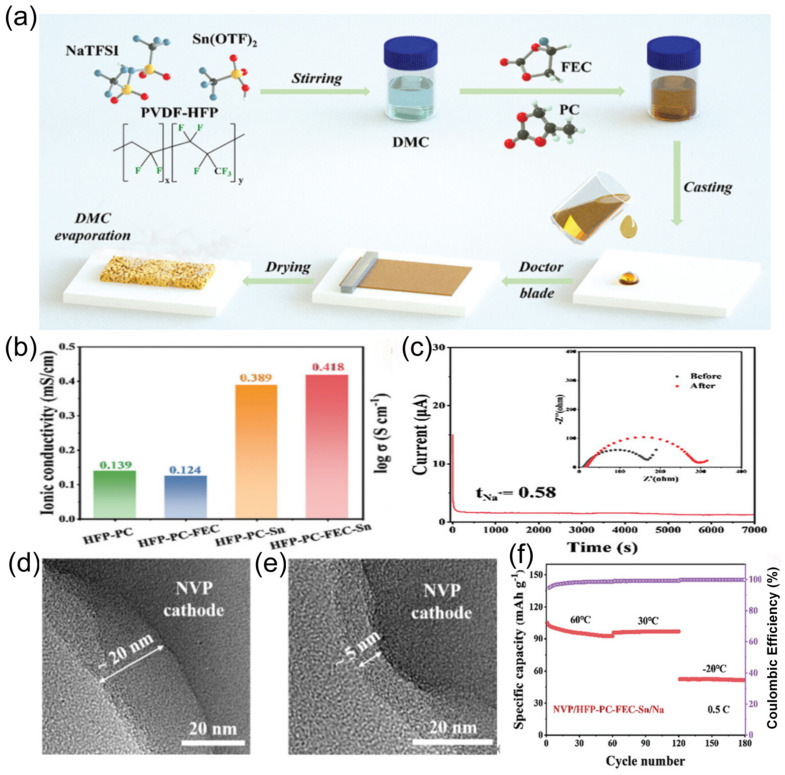
(**a**) Schematic illustration of HFP-PC-FEC-Sn QSPE. (**b**) Ionic conductivity of QSPEs. (**c**) Chronoamperometry curve of Na/HFP-PC-FEC-Sn/Na cells. The inset shows Nyquist plots before and after polarization. (**d**,**e**) TEM images of CEI film on an NVP cathode after 100 cycles. (**f**) Long cycling performance of NVP/HFP-PC-FEC-Sn/Na at 0.5 C. Copyright 2024 WILEY [131].

Yang et al. [131] combined a fluoroethylene carbonate (FEC) and tin trifluoromethanesulfonate (Sn(OTf)_2_) into the poly(vinylidenefluoride-co-hexafluoropropylene) (PVDF-HFP) and NaTFSI polymer matrix with a Sn(OTf)_2_ catalytic system and introduced PC and FEC into DMC solvent to construct a quasi-solid-state electrolyte (QSPE) with both high conductivity and interfacial stability (Figure 22a). Sn(OTf)_2_ catalyzes the ring-opening reaction of PC, reducing the transport barrier and enhancing sodium ion transport, endowing the prepared QSPE with an ionic conductivity of 0.42 mS cm^−1^ and a sodium ion transference number of 0.58 (Figure 22b,c). The reduction in desolvation energy remarkably reduced the interfacial reaction resistance. SEM images of Na anodes with BLTE (Figure 22d) and ES6-BLTE electrolytes (Figure 22e) after cycling revealed structural differences. When this electrolyte is applied to pouch batteries, it demonstrates stable performance at −20 °C (Figure 22f), not only inheriting the safety advantages of solid-state electrolytes but also compensating for the conduction defects of traditional solid-state materials through catalytic reactions.

**Figure 23:**
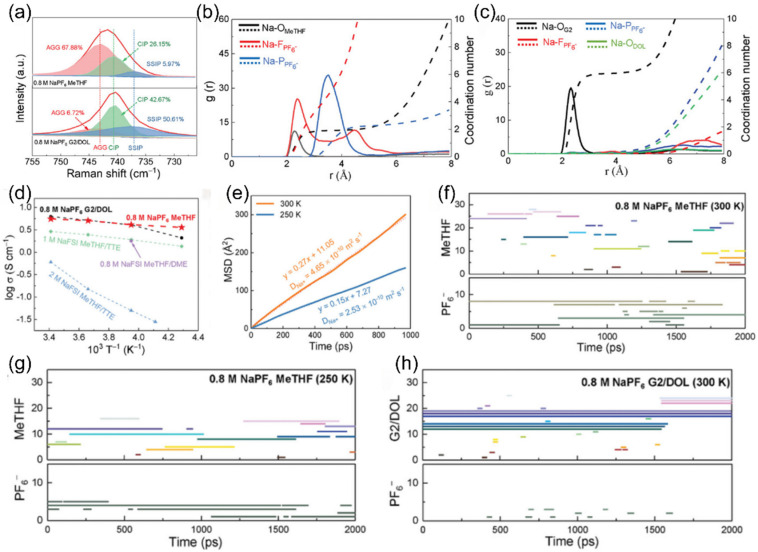
(**a**) Fitted Raman spectra of PF_6_^−^ anions, (**b**,**c**) Na^+^ RDF and coordination number, and (**d**) temperature-dependent ionic conductivity in the MeTHF and G2/DOL electrolytes. (**e**) The mean-squared displacements of Na^+^ in MeTHF at 25 and −25 °C. (**f**–**h**) Evolution of the Na-ion solvation environment in the MeTHF and G2/DOL. Copyright 2024 Wiley [132].

Ge team’s [132] diluted electrolyte with 2-methyltetrahydrofuran (MeTHF) as a key additive demonstrated ultra-high Coulombic efficiency for sodium plating/stripping at −25 °C and −40 °C (Figure 23a). Through a variety of characterization methods, it was found that the MeTHF additive induced the electrolyte to form an anion-π interaction-dominated solvation structure (Figure 23b,c). This structure not only endows anode-free sodium metal batteries (AFSMBs) with excellent ionic conductivity and charge conduction efficiency at low temperatures (Figure 23d,e), but also ensures the continuous and efficient operation of the charge transport network under low-temperature conditions through the stable architecture of ion conduction channels (Figure 23f–h).

On top of that, Jiang et al. [133] utilized the synergistic effect of heptafluorobutyramide (HFT) and lithium nitrate (LiNO_3_) to promote the dissolution of LiNO_3_, forming a robust SEI layer rich in Li_3_N/LiF. The He team [134] designed a quasi-ionic liquid additive [Li(15-crown-5)]NO_3_ (QIL), which induced the in situ generation of a highly ionically conductive Li_3_N-rich SEI, allowing the Li||Lithium Cobalt Oxide (LiCoO_2_) battery to cycle stably for 250 times without capacity decay at −20 °C. Zhang et al. [135] developed perfluoroalkylsulfonyl quaternary ammonium nitrate (PQA-NO_3_), whose cations formed an inorganic SEI and anions constructed a low-solvation structure. This allowed a 500 mAh soft-pack battery to retain 48.1% of its room-temperature capacity at −85 °C and achieve a high-rate discharge at 3.0 C under −50 °C.

Through the synergistic effects of solvent-based, film-forming, conductivity-enhancing, and stabilizing additives, the electrolytes can lower the freezing point, induce interfacial film formation, optimize ion transport, and suppress side reactions at low temperatures. By reconstructing the solventized structure of sodium ions, designing a quasi-solid electrolyte, introducing functional additives, and regulating the interfacial composition, the research teams effectively reduced the desolvation energy and interfacial impedance, inhibited the growth of sodium dendrites, and formed a highly ion-conducting and stable SEI (as shown in Table 3). These studies collectively indicate that for the low-temperature development of SIBs, the three-in-one strategy of “precisely regulating solvation structures, interfacial compositions, and ion conduction through additives” in lithium systems can be emulated. By combining the chemical properties of sodium metal/hard-carbon anodes, multifunctional additives with coordination groups can be designed to construct interfacial layers with both high ionic conductivity and mechanical stability, optimize the desolvation kinetics of Na^+^ ions, and inhibit dendrite growth, thus propelling the practical application process of low-temperature SIBs.

### 3.2. Optimization of Electrode Components and Structure

To enhance the low-temperature performance of SIBs from the perspective of synergistic modification of cathode and anode electrodes, it is necessary to optimize the Na^+^ diffusion kinetics within active materials and the ion migration mechanisms at the electrode/electrolyte interfaces according to the electrochemical behavior characteristics of different electrodes. In the design of cathode materials, the migration resistance of Na^+^ in the lattice at low temperatures mainly originates from the poor ion conduction pathways within the materials themselves and insufficient electronic conductivity.

#### 3.2.1. Cathode Modification

In the field of modifying cathode materials for low-temperature batteries, regulating the crystal structure can effectively shorten the diffusion path of Na^+^ and reduce the migration energy barrier. Coupled with the design of nanoparticles or surface carbon coating technology, it is possible to construct a continuous electronic conductive network, increase the contact area between the active material and the electrolyte, and enhance the interfacial charge transfer efficiency. Taking the layered oxide system as an example, optimizing the interlayer spacing and the occupancy of Na^+^ sites can improve the bulk ion transport kinetics. Combined with the modification of surface sodiumophilic groups, it can promote the uniform growth of the SEI film, forming a thin interfacial layer with excellent ionic conductivity.

**Figure 24:**
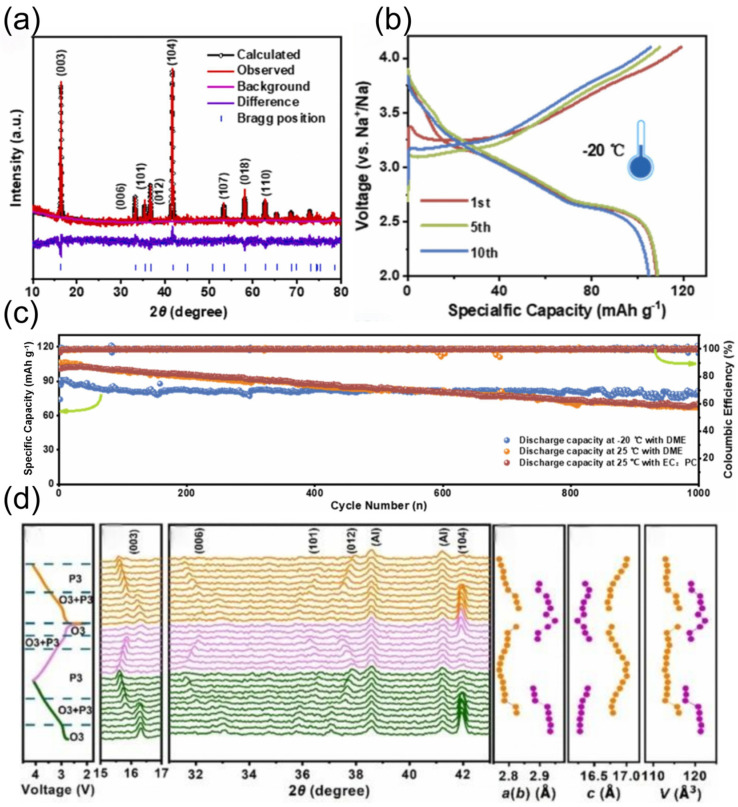
(**a**) Rietveld refinement of the XRD pattern for O3-FCNTMZ_1/12_. (**b**) Rate performance of FCNTMZ_1/12_ at 25 and −20 °C. (**c**) Long cycling performance of the FCNTMZ_1/12_ cathode. (**d**) In situ XRD patterns of O3-FCNTMZ_1/12_ at 25 °C. Copyright 2024 Elsevier [136].

The innovation of material modification strategies for SIBs always focuses on the improvement of ion transport kinetics and structural stability. Zeng et al. [136] developed the O3-FCNTMZ_1/12_ cathode material by regulating the energy levels of transition metals (Figure 24a) and controlling the difference in cation radius within 15%. This material achieved a reversible capacity of 109.6 mAh g^−1^ at 0.1 C under −20 °C (Figure 24b) and maintained 88% of its capacity after 1000 cycles at 1 C (Figure 24c). In situ XRD characterization revealed that the material underwent a reversible O3-P3 phase transition during charging and discharging, with a unit cell volume change of only 3% (Figure 24d). This provides a structural design for the low-temperature adaptation of layered oxide systems.

**Figure 25:**
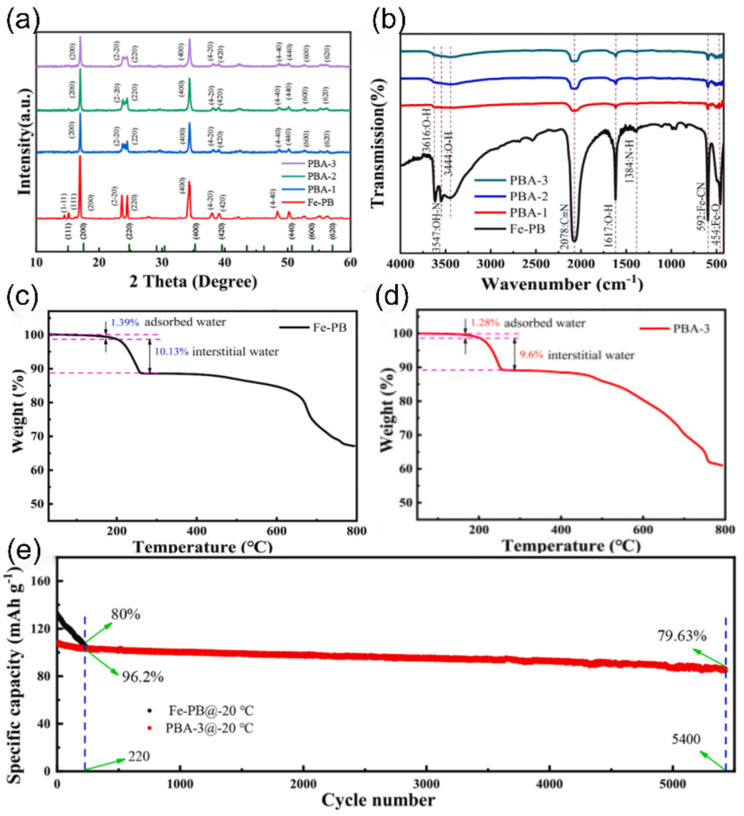
(**a**) XRD patterns of PBA with different Cs^+^ contents. (**b**) Fourier transform infrared spectroscopy (FTIR) spectra of Fe-PB and PBA-3. (**c**) TG curves of Fe-PB and (**d**) PBA-3. (**e**) The cycle capability of Fe-PB and PBA-3 at 5C at −20 °C. Copyright 2025 Elsevier [137].

In addition, Zhao et al. [137] proposed a Cs^+^/Zn^2+^ co-doping strategy to address the lattice stability and interface issues of iron-based Prussian blue analog cathodes. By using Zn^2+^ to support the lattice framework (Figure 25a) and Cs^+^ to stabilize the structure and reduce the content of interstitial water (Figure 25b–d), the cathode maintained 79.63% of its capacity after 5400 cycles at a high current density of 5C under −20 °C (Figure 25e). This strategy significantly suppressed structural collapse and electrolyte side reactions at low temperatures.

**Figure 26:**
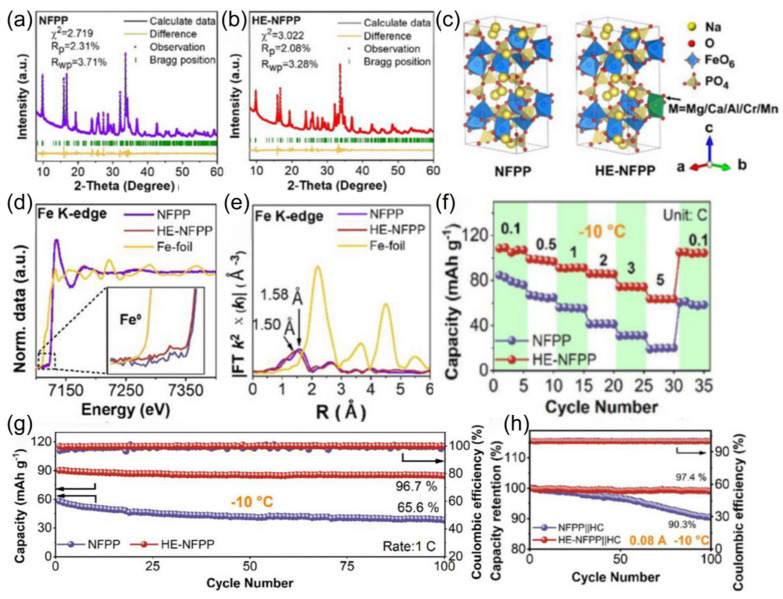
(**a**,**b**) Rietveld refinement of the XRD pattern, (**c**) the crystal structure, (**d**) the XANES spectra of the Fe K-edge, and (**e**) the FT-EXAFS of NFPP and HE-NFPP. (**f**,**g**) Rate performance from 0.1 to 5.0 C and cyclic performance under 1.0 C at −10 °C. (**h**) Discharge capacities and cyclic performance under 0.08 A at −10 °C. Copyright 2025 Wiley [138].

The application of the high-entropy strategy in the polyanionic Na_4_Fe_3_(PO_4_)_2_P_2_O_2_ (NFPP) cathode material involves introducing multiple transition metal ions [138]. The Rietveld refinement of XRD patterns for NFPP (Figure 26a) and Na_4_Fe_2.95_(MgCaAlCrMn)_0.01_(PO_4_)_2_P_2_O_7_ (HE-NFPP) (Figure 26b) reveals structural features. As depicted in Figure 26c, this aids in constructing 3D-network Na^+^-diffusion channels. The X-ray absorption near-edge structure (XANES) spectra (Figure 26d) and Fourier transform extended X-ray absorption fine structure (FT-EXAFS) (Figure 26e) of the Fe K-edge for NFPP and HE-NFPP disclose electronic and structural details. These modifications reduce the bandgap, significantly enhancing electron conduction and ion diffusion efficiency, while suppressing volume changes during charging and discharging. After modification, the material exhibits a capacity-retention rate of 96.7% after 100 cycles at 1.0 C under −10 °C (Figure 26g). The rate performance from 0.1 to 5.0 C is illustrated in Figure 26f. This breakthrough overcomes the low-temperature kinetic limitations of traditional polyanion materials, highlighting the advantages of synergistic multi-component modification.

In the field of LIBs, the antimony doping [139] strategy addresses the H2-H3 phase transition challenge of the high-nickel layered oxide LiNi_0.91_Co_0.06_Al_0.03_O_2_. By constructing a radially arranged microstructure, it disperses anisotropic mechanical stress, suppresses microcrack generation, and optimizes the lithium-ion transport pathway. As a result, the material maintains 84% of its capacity after 200 cycles at 1C within the voltage range of 2.7–4.5 V at 25 °C. At −20 °C, the capacity retention rate increases from 61% to 88% after 100 cycles at 0.5 C, providing a new structural regulation idea for the low-temperature adaptation of high-voltage and high-nickel systems. Regarding the NASICON-type Li_3_V_2_(PO_4_)_3_ cathode, the C+YPO_4_ [140] binary surface coating strategy suppresses the decomposition of the electrolyte under high voltage, forming a thin and stable solid electrolyte interface film. This strategy enhances the lithium-ion diffusion coefficient to the order of 10^−6^ cm^2^ s^−1^. At −20 °C and −40 °C, the capacity retention rates reach 89.1% and 75.7%, respectively. Even at an ultra-high rate of 50 C, it still maintains rapid ion transport, offering an efficient solution for the kinetic optimization of cathodes in SIBs under low-temperature environments.

With deeper research, low-temperature solid-state SIBs have made breakthroughs in electrode materials. Xu et al. [141] developed a solid-state self-assembled flaky Na_3_V_2_(PO_4_)_3_@carbon spherical superstructure (SS-NVP@C) cathode material. This innovative material achieves a discharge capacity of 92 mAh g^−1^ in a low-temperature solid-state sodium-ion battery at −40 °C, with a rate performance of 51 mAh g^−1^ at a 5C discharge rate. Additionally, the capacity retention rate reaches 84.8% after 400 cycles, offering a promising approach for low-temperature energy storage systems.

These strategies, ranging from atomic-level doping and nanostructure design to interface engineering, have systematically broken through the core bottlenecks of slow ion transport, unstable interface, and structural degradation at low temperatures. Whether it is the regulation of reversible phase transitions in the O3-FCNTMZ_1/12_ cathode material for SIBs, the stabilization of Prussian blue analogs through dual-ion doping, or the radial microstructure design enabled by antimony doping and the interfacial optimization via C+YPO_4_ coating in LIBs, they all achieve performance enhancements of low-temperature batteries through multi-dimensional approaches involving “lattice optimization-interface modification-conduction pathway design”. The design of conjugated structures in polymer cathodes shows the unique advantages of organic materials, offering a flexible solution for low-temperature batteries.

These advancements not only strengthen the theoretical foundation of the synergistic optimization of “structure interface kinetics” but also push battery materials towards full temperature range applications, providing crucial technical support for energy storage in extreme environments. This marks that low-temperature battery materials are moving towards the stage of engineering from the optimization of a single strategy to the integration of multidisciplinary engineering.

#### 3.2.2. Anode Electrode Modification

In low-temperature environments, the migration resistance of Na^+^ at the electrode/electrolyte interface is the core bottleneck restricting the performance of anode electrodes, primarily manifested as an increase in the resistance of the SEI film and the passivation of active sites. In response to different types of anode electrode materials, researchers have developed differentiated low-temperature performance optimization strategies through precise interfacial engineering and structural regulation, forming a systematic solution ranging from mechanism innovation to material design.

**Figure 27:**
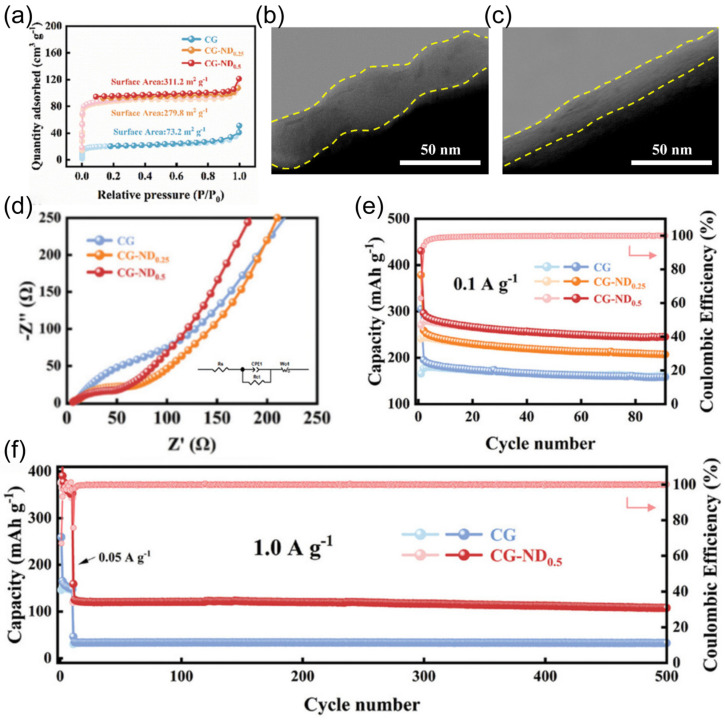
(**a**) Nitrogen adsorption/desorption isotherm curves. (**b**,**c**) TEM images after charge/discharge cycles. (**d**) Nyquist plot after 90 cycles. (**e**) Cycle performance of 90 cycles. (**f**) Cycle performance of 500 cycles at 1.0 A g^−1^. Copyright 2025 Wiley [142].

For carbon-based anodes, the core of modification lies in the synergistic regulation of pore structure and surface chemistry to construct an efficient interfacial microenvironment conducive to ion transport. Zhao et al. [142] introduced nanodiamonds (NDs) into cattail-based hard-carbon anodes. Leveraging the high hardness and surface activity of NDs to induce microporous and disordered carbon structures to bring the specific surface area up to 311.2 m^2^ g^−1^ (Figure 27a), thus providing abundant reversible adsorption sites for Na^+^. Meanwhile, the strong interaction between the functional groups on the ND surface and Na^+^ reduced the interface resistance to 22.1 Ω (Figure 27d). Combined with the inhibition effect of the nanoscale support on the volume contraction, a thin and dense SEI film was formed at −40 °C (Figure 27b,c), effectively inhibiting electrolyte decomposition and sodium dendrite growth. As a result, it achieved a reversible capacity of 245.1 mAh g^−1^ after 90 cycles at 0.1 A g^−1^ (Figure 27e) and maintained a capacity retention rate of 90% after 500 cycles at 1.0 A g^−1^ (Figure 27f).

**Figure 28:**
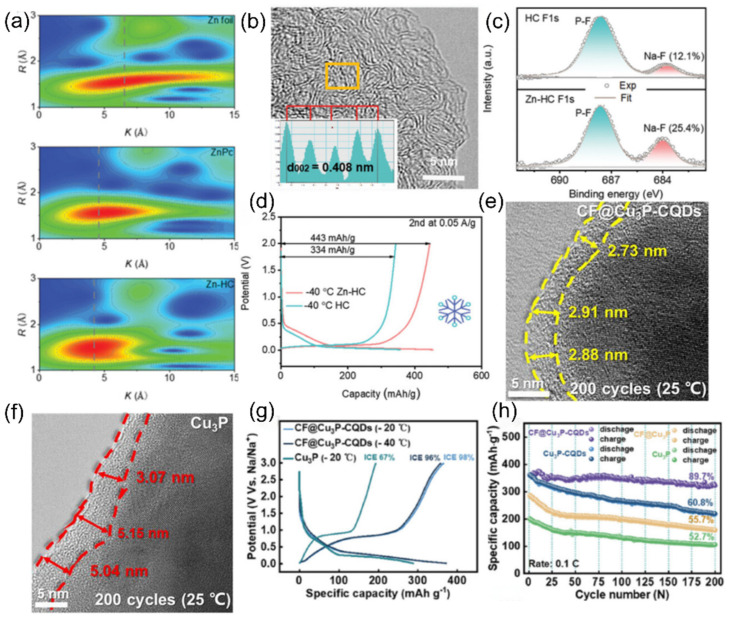
(**a**) Wavelet transform of the k2-weighted EXAFS data. (**b**) HRTEM image of Zn-HC. (**c**) XPS spectra of F 1s of the SEI layers on Zn-HC and HC. (**d**) The GCD curves. Copyright 2023 Wiley [78]. (**e**,**f**) TEM images of CF@Cu_3_P-CQDs and Cu_3_P after 200 cycles. (**g**) Initial discharge/charge profiles at 0.1 C. (**h**) Cycling stability at 0.1 C. Copyright 2025 Wiley [144].

On this basis, Lu et al. [78] employed atomic-level zinc single-atom doping to construct a Zn-N_4_-C coordination structure, which induced a local electric field (Figure 28a). This approach effectively expanded the interlayer spacing of graphite to 0.408 nm (Figure 28b) and optimized the distribution of nanopores. Meanwhile, it synergistically catalyzed the decomposition of NaPF_6_ in the electrolyte, leading to the formation of a thin, NaF-rich inorganic SEI film (Figure 28c). As a result, the interfacial charge transfer resistance and the diffusion barrier of sodium ions in the bulk phase were significantly reduced. This enabled the hard-carbon anode to achieve a high discharge capacity of 443 mAh g^−1^ and an 85% cycle retention rate at −40 °C (Figure 28d).

Similarly, the Issatayev team [143] utilized the synergistic effect of LiF surface modification and nitrogen doping to construct a 6 nm-thick LiF-rich thin SEI film on the surface of the hard carbon. Combined with a porous structure with pore sizes of 0.8–1.2 nm, this strategy accelerated the desolvation of lithium ions and interfacial charge transfer. Consequently, the material delivered a capacity of 280 mAh g^−1^ after 100 cycles at 0.1C and maintained 72 mAh g^−1^ after 500 cycles at a high rate of 2 C under −20 °C. These results prove that the synergistic effect of interfacial composition regulation and pore structure optimization can effectively overcome the low-temperature kinetic limitation of carbon-based materials.

For alloy-based and novel compound anodes, low-temperature modification strategies primarily focus on nano-confinement, defect engineering, and composite structure design. For example, nitrogen-doped carbon quantum dots (CQDs)-modified Cu_3_P nanoparticles anchored on carbon fiber anodes (CF@Cu_3_P-CQDs) [144] were synthesized via a green method. The CQDs regulated interfacial contact and facilitated the uniform formation of the SEI film (Figure 28e,f), significantly improving electron transfer efficiency. At −40 °C, the CF@Cu_3_P-CQDs anode achieved a capacity of 350 mAh g^−1^ at 0.1C (Figure 28g) and retained 88.5% of its capacity after 100 cycles (Figure 28h), demonstrating that nanoscale interfacial modification can effectively suppress the volume expansion of alloy-based materials.

**Figure 29:**
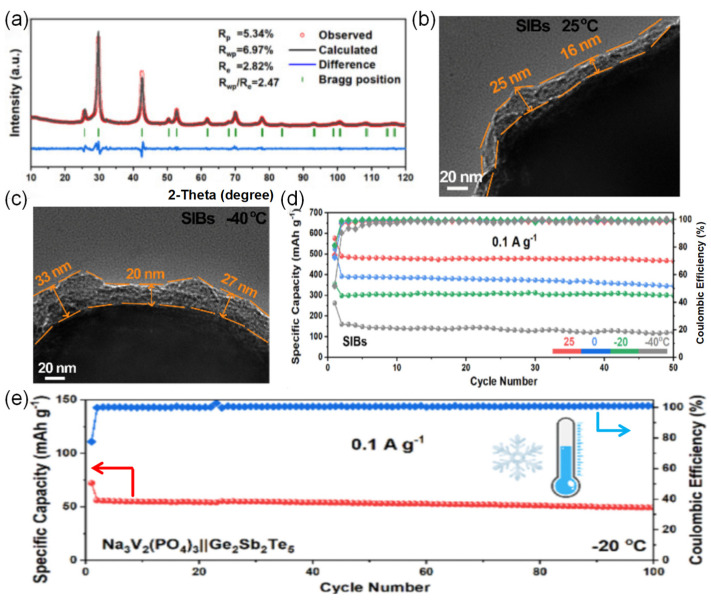
(**a**) Rietveld refinement of the XRD pattern. (**b**,**c**) Ex situ TEM images after 50 cycles of Ge_2_Sb_2_Te_5_ at 25 °C and −40 °C for SIBs. (**d**) Rate capabilities of Ge_2_Sb_2_Te_5_ SIBs. (**e**) Galvanostatic charge/discharge profiles of the Ge_2_Sb_2_Te_5_||NVP full-cell. Copyright 2025 Elsevier [145].

Zhao’s team [145] developed a Ge_2_Sb_2_Te_5_ cubic-crystal anode material with enriched cation vacancies (Figure 29a). By leveraging the electronic structure reconstruction of germanium/antimony vacancies and tellurium atoms, this material increases the exposure of active sites and induces the formation of an inorganic NaF-rich SEI film (Figure 29b,c). In the half-cell of a sodium-ion battery at −40 °C, it achieved a discharge capacity of 161 mAh g^−1^ (Figure 29d). The full-cell demonstrated an energy density of 149 Wh kg^−1^ at −20 °C, with a capacity retention rate of over 90% after 100 cycles (Figure 29e). This research reveals the triple mechanisms by which atomic-level defects optimize electronic conductivity, ion adsorption, and interfacial stability.

In the field of LIBs, Jiang et al. [146] designed a composite anode of polypyrrole (PPy)-coated solid-state molten salt–iron oxide (SMS-Fe_2_O_3_@PPy). The conductive network of PPy suppresses volume expansion, while the strong adsorption of Li^+^ by the Fe(NMP)_6_^2+^ active sites in the solid-state molten salt enables a capacity retention of 1198 mAh g^−1^ after 1000 cycles at 1C and a capacity of 530 mAh g^−1^ at a high rate of 2 C under −20 °C. This demonstrates that the synergistic effect of conductive polymers and active sites can effectively inhibit interfacial side reactions and enhance structural stability at low temperatures. Pogue’s team [147] prepared Nb_2_O_5−x_ anode material with a Wadsley–Roth structure through oxygen defect engineering and nanoparticle size optimization. Its unique crystal structure shortens the diffusion path of Li^+^, and the interfacial defects induce the formation of a highly conductive SEI film. In a full battery at −20 °C, when paired with an LMO cathode, it maintains a capacity of 90 mAh g^−1^ at a 2 C rate, with a capacity retention of 98% after 50 cycles. Moreover, under the simulated high-frequency load conditions of drones, the voltage decay is only 0.3 V, providing an anode electrode design paradigm of “defect structure” synergistic optimization for low-temperature, high-power scenarios.

These studies indicate that, despite the differences in the physical and chemical properties of sodium and lithium ions, the core logic for enhancing their low-temperature performance is highly consistent—namely, breaking through the bottlenecks of mass and heat transfer through cross-scale synergy in interfacial phase regulation, bulk phase structure optimization, and conduction collaborative design. The advancements in LIBs, such as the rapid ion channels induced by oxygen defects and the buffer mechanism of conductive polymers against volume changes, in aspects like defect regulation and composite interface design, provide direct methodological references for SIBs. This cross-system knowledge transfer emphasizes the universality of optimization strategies and paves the way for further technological convergence between the two battery systems.

### 3.3. Electrode/Electrolyte Interface Optimization

In the field of low-temperature batteries, interfacial optimization stands as the core strategy to overcome the bottlenecks of ion transport kinetics and interfacial stability issues. Through multiple approaches such as electrolyte engineering, structural design, and catalytic engineering, it is possible to effectively regulate the chemical composition and microstructure of the interface, accelerate ion desolvation and transport at low temperatures, suppress side reactions, and buffer volume changes. In the realm of low-temperature battery interfacial optimization, diverse strategies are pushing the sodium-ion system to break through the performance limit.

**Figure 30:**
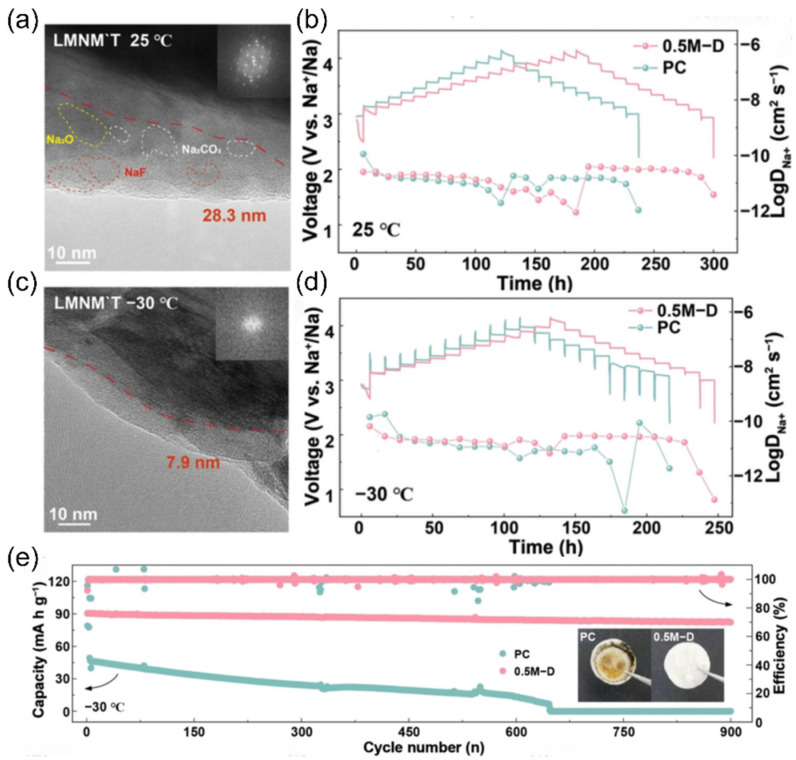
(**a**,**c**) The Cryo-TEM images of LMNM’T cycled at 25 and −30  °C. (**b**,**d**) GITT curves and the calculated Na^+^ diffusion coefficient of LMNM’T//Na at 25 °C and −30  °C. (**e**) The cycling performance of LMNM’T half-cells at 1C under −30  °C. Copyright 2024 Wiley [148].

For SIBs, the core of interfacial regulation lies in constructing a homogeneous, low-resistance, and low-temperature-resistant reaction layer. Feng et al. [148] employed a low-concentration electrolyte system with 0.5 M NaPF_6_ dissolved in diglyme (DG), successfully creating a thin and uniform amorphous single electrode/electrolyte interfacial layer at low temperatures (Figure 30a,c). This thin and homogeneous amorphous interfacial layer formed on the electrode surface features a synergy between flexible organic components and amorphous inorganic components, which reduces the migration hindrance of Na^+^, effectively suppressing side reactions between the active material and the electrolyte and significantly enhancing the stability of the interfacial structure. Such highly homogeneous interfacial properties accelerate the interfacial transport kinetics of Na^+^ at low temperatures (Figure 30b,d), endowing the battery with excellent low-temperature performance: the LMNM’T/Na half-cell maintains a capacity retention rate of 90.8% after 900 cycles at 1 C under −30 °C (Figure 30e).

**Figure 31:**
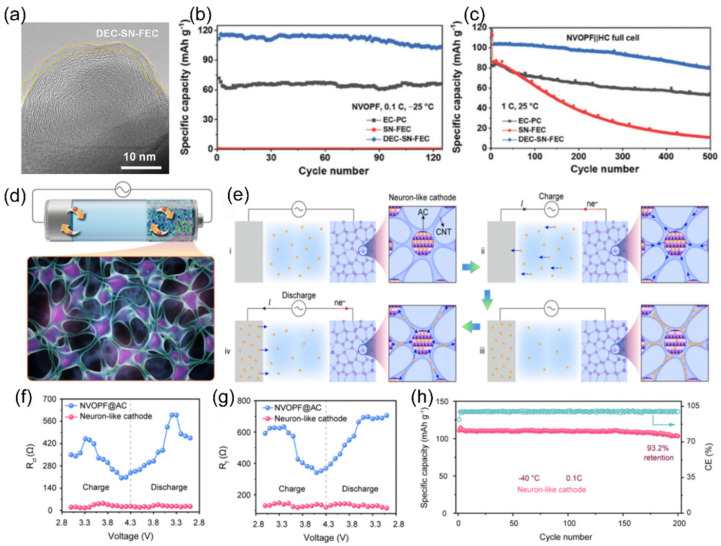
(**a**) CEI layers on NVOPF after 300 cycles. Cycling performance of (**b**) NVOPF and (**c**) NVOPF||HC full-cells under 25 °C. Copyright 2023 Wiley [149]. (**d**) Schematic illustration of the bioinspired battery containing the neuron-like cathode. (**e**) Detailed process of Na^+^ transport. Ion transport confinement originates from Na+ ions diffuse rapidly from cathode particles to CNTs (from i to ii), and CNTs subsequently trap Na+ ions released from the particles during charge (iii). The AC layer facilitates the migration of Na+ ions on the particle surface into CNTs. Na+ ions return directly from CNTs to particles during discharge (from iii to iv), thereby eliminating sluggish charge transfer at the cathode-electrolyte interface. (**f**,**g**) Evolution of R_ct_ and R_f_ forNVOPF@AC and neuron-like cathodes. (**h**) Long-term cycling stability and corresponding CE at −40 °C. Copyright 2024 American Chemical Society [150].

The research by Liu’s team [149] focused on constructing a stable interface for high-voltage SIBs through solvent engineering. They used a combination of succinonitrile (SN) and diethyl carbonate (DEC) as co-solvents, synergizing with fluoroethylene carbonate (FEC) to form an ultra-thin and uniform nitrogen/fluorine-rich interfacial layer (Figure 31a). With the high-pressure resistance property of SN to generate Na_3_N- and NaF-rich CEI on the surface of the cathode electrode, combined with the low freezing point (−74.3 °C) of DEC and the interfacial strengthening effect of FEC, the NVOPF half-cell achieved a capacity retention of 92% at −25 °C (Figure 31b), and the full-cell maintained 95.3% of its capacity after 100 cycles (Figure 31c).

The design of bionic structures and heterointerfaces further expands the low-temperature adaptability of sodium batteries. Inspired by the signal transmission of neurons, Huang et al. [150] utilized carbon nanotubes (CNTs) to construct a solid-state ion transport network (Figure 31d,e). This network confines the sodium ions released during charging within the internal network of the cathode, completely avoiding charge transfer at the solid/liquid interface. The amorphous carbon (AC) coating shortens the ion transport path, forming an efficient solid-state conduction channel, which reduces the interfacial charge transfer resistance by 14 times (Figure 31f,g). Under low-temperature conditions, this design breaks through the limitations of the electrolyte, achieving a capacity retention rate of 93.2% at 0.1 C under −40 °C (Figure 31h).

**Figure 32:**
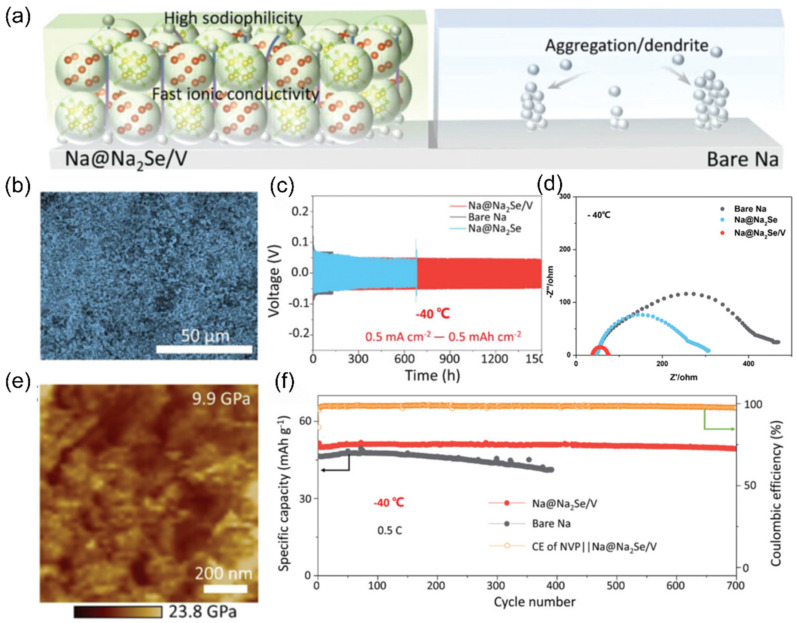
(**a**) Schematic illustration of Na^+^ plating. (**b**) SEM image of the Na@Na_2_Se/V electrode. (**c**) Electrochemical performance at −40 °C. (**d**) EIS curves under −40 °C. (**e**) Young’s modulus of the Na@Na_2_Se/V protective layer. (**f**) Cycling performance at −40 °C. Copyright 2022 Wiley [151].

Xia and his colleagues [151] successfully constructed an artificial heterogeneous interfacial layer composed of sodium diselenide (Na_2_Se) and vanadium (V) metal on the surface of sodium metal through an in situ chemical reaction. This layer leverages the high sodophilicity, excellent ionic conductivity, and high Young’s modulus of the materials to enhance the adsorption and transport of sodium ions, resulting in homogeneous and dendrite-free deposition. Additionally, it accelerates the desolvation of solventized sodium ions at −40 °C. A schematic illustration of Na^+^ plating on bare Na and Na@Na_2_Se/V electrodes is presented in Figure 32a. The combination of these two materials forms an interfacial layer with both electrical insulation and high mechanical strength (Figure 32b,e), effectively buffering the volume changes during sodium deposition and stripping. At low temperatures, this interfacial layer leverages the superionic conductivity of Na_2_Se and the high sodium affinity of V to accelerate the desolvation of solvated sodium ions. As a result, at −40 °C, the symmetric cell can cycle for over 1500 h at a current density of 0.5 mA cm^−2^ (Figure 32c) with a voltage hysteresis of only 20 mV. After the successful formation of the Na_2_Se/V heterointerface, the impedance is drastically reduced, as shown in Figure 32d. When paired with a Na_3_V_2_(PO_4_)_3_ cathode in a full-cell, it maintains 86.5% of its capacity after 700 cycles at 0.5 C, with a Coulombic efficiency approaching 100% (Figure 32f).

**Figure 33:**
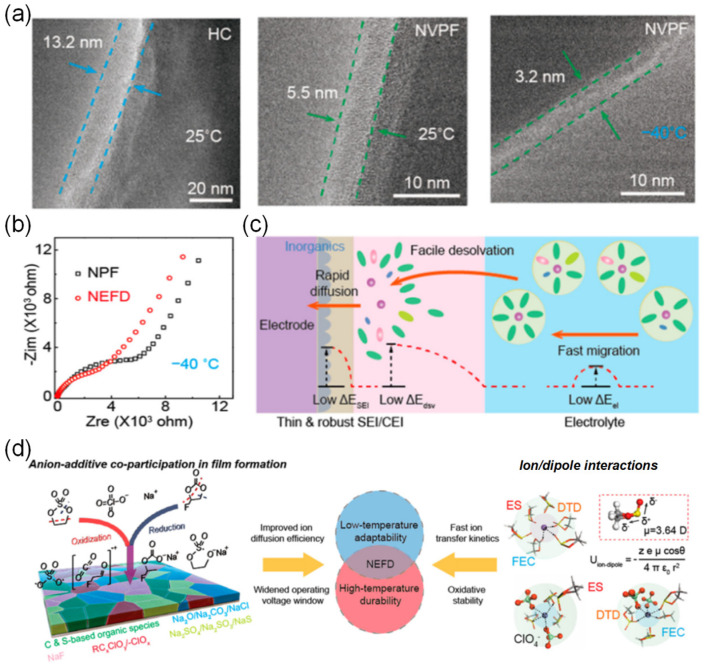
(**a**) HRTEM with morphological characterization for SEIs (HC) and CEIs (NVPF) cycled in the NEFD electrolyte. (**b**) Comparison of EIS spectra at low temperature. (**c**) Ion-transfer processes and associated energy barriers in the electrolyte and at the interface. (**d**) Schematic representation of solvation design and interphase modulation. Copyright 2025 American Chemical Society [152].

In the latest study, Liang et al. [152] proposed, for the first time, an electrolyte system based on cyclic sulfite, which accelerates the desolvation process by precisely regulating the ion/dipole interactions and directionally screening the dominant ion coordination states. With the aid of synergistic decomposition of the anions and additives, the SEI/CEI interfacial layer is formed at the electrode interface. This layer is rich in inorganic components, robust, and exhibits excellent ion diffusion capabilities. The HRTEM image of Figure 33a confirms that the thickness of the interfacial layer at a low temperature is only 3.2 nm, and it is both uniform and dense. The low-temperature impedance spectra of Figure 33b demonstrate that the interfacial resistance of the system is significantly lower than that of conventional electrolytes. Figure 33c further reveals that the “inorganic inner layer + organic outer layer” structure of the interfacial layer reduces the ion diffusion barrier and enhances the thermal stability. Additionally, Figure 33d highlights the synergistic effect of the solvent-based structural design and interfacial modulation achieved through the optimization of ion/dipole interactions and the synergistic decomposition of anion additives, resulting in rapid ion transport and a thermally stable interface across a wide temperature range.

In the field of LIB interface optimization, Liang et al. [153] focused on the challenge of co-intercalation of electrolytes in graphite anodes. By introducing the inorganic anion DFP, they leveraged its stronger coordination ability with Li^+^ to regulate the solvation structure, leading to an SEI layer rich in highly ion-conductive Li_3_PO_4_ and reducing the expansion rate. This interfacial layer effectively suppressed the side reactions caused by the intercalation of electrolyte components into the interlayer of graphite at low temperatures, overcoming the problem of sudden capacity drop caused by the instability of the SEI in traditional carbonate electrolytes. Min’s team [154] further expanded the concept of solvent engineering by constructing anion-rich solvation sheaths in strong solvents. This approach promoted the formation of an inorganic-rich interfacial layer on the electrode surface, containing B-O, B-F, and LiF components. This interfacial layer exhibits both low impedance and high stability, not only accelerating the desolvation process of Li^+^ at low temperatures but also maintaining the integrity of the interface structure by suppressing parasitic reactions.

Although these two research efforts adopted different approaches, their core focus remained on “inorganic modification of the interface and enhancement of ionic conductivity”. Together, they provided solutions ranging from mechanism exploration to engineering implementation for the efficient and stable operation of graphite-based anodes at low temperatures, thereby promoting the breakthrough of SIBs in low-temperature scenarios.

The key strategies derived from the research on low-temperature interface optimization of LIBs offer valuable references for solving the interface challenges of low-temperature SIBs. Although both types of batteries face interface challenges, such as electrode expansion caused by the co-intercalation of solvents and increased impedance of the SEI film at low temperatures, the interface issues of SIBs are more complex and severe in low-temperature environments due to the larger ionic radius and solvation energy of sodium ions. Based on the core concept of “inorganic modification of the interface and enhancement of ionic conductivity”, inorganic anions with strong coordination ability can be introduced to regulate the solvation structure of sodium ions. This approach breaks the tight encapsulation of Na^+^ by solvent molecules, promoting the formation of a thin SEI film rich in inorganic components such as NaF and Na_3_PO_4_ on the electrode surface. This not only reduces interfacial impedance but also effectively suppresses the co-intercalation behavior of solvents. Meanwhile, functional compounds containing B-O and B-F groups can be added to the electrolyte to promote the formation of a composite protective layer with both high ionic conductivity and mechanical stability at the interface. This protective layer can accelerate the desolvation kinetics of Na^+^ at low temperatures and maintain the integrity of the interface structure by inhibiting side reactions of electrolyte decomposition.

## 4. Challenges and Outlook

### 4.1. Challenges

The core challenges faced by low-temperature SIBs in low-temperature environments can be summarized in the following four points: (1) The viscosity of the solvent rises significantly at low temperatures, leading to an increase in sodium ion migration resistance, while the solidification point of the solvent restricts the temperature domain of the electrolyte liquid phase, which is prone to triggering local polarization or even solidification failure. Compared with carbonate solvents commonly used in LIBs, SIBs can use ether solvents or low-freezing-point co-soluble systems, which have a 30–50% lower increase in viscosity with temperature than LIBs, and the liquid-phase window can be extended to below −50 °C, which is significantly superior to the −30 °C limit of LIBs. In addition, an insufficient dielectric constant and coordination ability of the solvent will weaken the dissociation efficiency of the sodium salt, forming a tight solvation sheath layer, which further raises the desolvation energy barrier of sodium ions. Thanks to the weaker Lewis acidity of Na^+^ compared to Li^+^, the binding energy of the solvated sheath layer of SIBs is 20% to 40% lower than that in LIBs. This difference provides an inherent advantage for optimizing the solvation energy barrier at low temperatures. It is necessary to synergistically optimize the ion transport kinetics and interfacial stability by designing solvent systems with low viscosity, a low freezing point, and a wide electrochemical window. (2) The decrease in dissociation of sodium salts at low temperatures directly reduces the effective carrier concentration of the electrolyte, while the strong coordination of anions with the solvent may exacerbate the sodium ion transport hysteresis. In contrast, while the lithium salts used in LIBs, such as LiPF_6_, are more dissociated, their anions are prone to form stable coordination structures with the solvent at low temperatures. This interaction leads to a Li^+^ migration number that is even lower than the Na^+^ migration number observed in SIBs. At the same time, the insufficient chemical stability of sodium salts can lead to the generation of harmful by-products, which corrode the electrode interface and damage the integrity of the interfacial membrane. Through anionic structure modulation, SIBs can construct anion-derived interfacial membranes that are more stable than LIBs, with an electrochemical window that can be broadened to above 4.5 V, close to the level of LIBs. Modulation of the anionic structure of sodium salts is needed to enhance the dissociation ability, broaden the electrochemical window, and inhibit side reactions and dendrite growth. (3) The lattice shrinkage and blocked ion diffusion channels of anode materials significantly increase the activation energy of sodium ion migration, while the low intrinsic electronic conductivity exacerbates the low-temperature polarization; the retarded sodium ion adsorption/desorption kinetics and surface defects of anode materials easily lead to inhomogeneous sodium deposition. In contrast to the severe Li^+^ diffusion retardation and dendrite growth of LIB graphite anodes at low temperatures, the layer spacing of the SIB hard-carbon anode is larger than that of graphite, and the surface defect sites can be transformed into Na^+^ fast transport channels by the pre-sodiation treatment. This results in a sodium ion diffusion coefficient at −40 °C of 10^−10^ cm^2^ s^−1^, which is one order of magnitude higher than that of LIBs graphite anode under the same conditions. In addition, the volume expansion and contraction of electrode materials during the sodiation/desodiation process can easily trigger particle pulverization or interface stripping, accelerating the capacity decay. Through crystal structure modulation, the volume change rate of the anode in SIBs can be controlled within 3%, which is significantly lower than that of the NMC anode in LIBs, which is 6–8%, and thus enhances the mechanical stability. It is necessary to enhance the ion diffusion rate and mechanical stability of the materials through crystal structure modulation, surface modification, and pore engineering. (4) The desolvation process of sodium ions at low temperatures requires overcoming higher energy barriers, and insufficient ionic conductivity of the interfacial membrane further increases the interfacial impedance. Unlike the thick and highly resistive SEI membranes formed by LIBs at low temperatures, SIBs can construct ultrathin, high ionic conductivity interfacial membranes with additives, and their low-temperature impedance can be reduced to below 50 Ω cm^2^. At the same time, electrode volume changes and electrolyte decomposition can easily lead to rupture of the interfacial film, triggering continuous interfacial remodeling and accumulation of side reactions. Since the Stokes radius of Na^+^ is smaller than that of Li^+^, the Na^+^ migration activation energy of the interfacial membranes of SIBs is lower than that of Li^+^ of LIBs, which results in the interfacial ion transport efficiency at −30 °C improving by more than 40%. Thin and dense interfacial films with high ionic conductivity and mechanical stability need to be constructed through electrolyte additives or artificial interfacial layer design to reduce desolvation energy and inhibit interfacial degradation.

### 4.2. Outlook

Future development of low-temperature SIBs will require innovations in solvents, sodium salts, electrodes, and interfaces in the electrolyte.

(1) In the future, we can focus on developing new solvent systems with low viscosity, a low freezing point, and a wide electrochemical window. Through the introduction of ethers, fluorinated esters, and other solvents or the design of multi-component blending systems, combined with artificial intelligence-assisted molecular simulation and high-throughput screening technology, the solvent ratio can be optimized quickly to reduce the resistance to migration of sodium ions and inhibit low-temperature crystal precipitation. At the same time, we explore the strategy of ionic liquids or local high-concentration electrolytes to regulate the solvation structure at the molecular scale, balance the ionic conductivity and interfacial stability, and provide a new path for efficient ion transport at low temperature.
(2) To address the dissociation kinetics and interfacial compatibility of sodium salts, we will focus on anion structure innovation in the future to enhance the low-temperature dissociation capability by weakening ion-pair interactions. At the same time, we will study the sodium salt concentration gradient electrolyte or double-salt system to widen the electrochemical window and inhibit the growth of dendrites by using the synergistic effect of anions. Combined with in situ spectroscopy to analyze the coordination behavior of sodium salt solvent, a dynamic model of “dissociation–migration–interfacial reaction” is established to guide the molecular design of sodium salt and the optimization of electrolyte formulation.
(3) Cathode electrode materials will be developed towards highly stable open framework structures, and the low-temperature ion diffusion rate and electronic conductivity will be enhanced by lattice doping or surface coating. For anode materials, a combination of hard-carbon pore modulation, alloy nanosizing, and pre-sodiation strategies are needed to lower the sodium ion embedding barrier and mitigate volume expansion. In addition, machine learning will accelerate the screening of high-performance materials and combine with material genomics to build a low-temperature suitability database. In recent work on combining artificial intelligence with energy storage technologies, Chen et al. [155] constructed the Uni-Electrolyte platform through artificial intelligence, which integrates the EMolCurator (molecular design), EMolForger (synthetic pathway prediction), and EMolNetKnittor (interfacial reaction analysis) modules. This platform enables efficient design, synthetic planning, and analysis of the interfacial mechanisms of rechargeable battery electrolyte molecules. Meng et al. [156] constructed a deep learning model of the MBVGNN graph through artificial intelligence, which integrates global and geometric information to accurately predict the average voltage, formation energy, and other properties of cathode materials for SIBs. This model efficiently screened over 70,000 high-entropy and fluorine-substituted materials, providing 16 material types and more than one million element combinations for reference in experimental preparation. This approach significantly accelerates the research and development of high-performance battery materials. Together, these studies have promoted the closed-loop development mode of “theoretical prediction–experimental verification”.
(4) Future interfacial optimization will focus on building thin, dense SEI/CEI membranes with high ionic conductivity. Inorganic-rich interfacial films are induced by electrolyte additives to reduce the desolvation energy barrier and inhibit the continuous decomposition of the electrolyte. Further development of atomic layer deposition (ALD) or molecular layer deposition (MLD) technology to construct an artificial interfacial layer on the electrode surface to achieve a dynamic balance between interfacial mechanical strength and ionic conductivity. At the same time, adaptive interfacial repair strategies should be developed, and functional additives should be used to repair interfacial defects dynamically during the cycling process so as to enhance the stability of low-temperature and long-cycle electrodes.

## 5. Summary

In the context of global energy transitions, SIBs have become an important direction for energy storage in extreme environments due to their resource advantages and low-temperature adaptability. In this review, we systematically discuss the three core bottlenecks of the performance degradation of low-temperature SIBs: (1) a significant increase in the viscosity of the electrolyte at low temperatures resulting in a sudden drop in the ion conduction efficiency, (2) a deterioration in the structural stability of the electrode material caused by lattice distortion and phase transition, and (3) an increase in the interfacial desolvation barrier accompanied by a decrease in the densification of the SEI/CEI membrane, leading to an increase in the resistance of charge transfer. To address the above problems, the study proposes a three-pronged optimization strategy: (1) based on low-freezing-point solvent compounding, sodium salt molecular design, and functional additives to synergistically regulate the solvation structure of the electrolyte to enhance the ion mobility rate and the interfacial stability, (2) optimize the crystalline structure of the electrode material and ion diffusion kinetics through atomic-level doping modification, nano-restricted structure construction, and biomimetic transport network design, (3) optimize the crystal structure of the electrode material and ion diffusion kinetics; with the help of artificial heterogeneous interfacial layer construction, temperature-adaptive solvation structure regulation and catalytic desolvation mechanisms, a low-impedance and high-stability electrode/electrolyte interface is constructed, which provides a systematic solution to improve the performance of sodium-ion batteries in extreme low-temperature environments.

Recent advances in hierarchical doping—spanning bulk-phase, surface, and interfacial modifications—provide a cohesive strategy to address these challenges. Combined with in situ characterization and theoretical simulations, these studies reveal that hierarchical doping fundamentally alters solvation shell dynamics and interfacial film evolution, offering a unified framework to counteract performance degradation. Future efforts must prioritize the synergistic optimization of electrolyte/electrode interface systems through multiscale doping architectures. Such innovations could enable the development of electrolyte formulations and electrode materials specifically engineered for extreme environments, ultimately accelerating the deployment of SIBs in polar and deep-sea applications. By bridging atomic-scale doping effects with macroscopic performance, this approach paves the way for climate-resilient, high-efficiency energy storage technologies. Finally, we present here a table comparing the strategies in this paper in terms of key parameters such as initial capacity, capacity retention, and operating temperature (as shown in Table 4).

## Figures and Tables

**Table 1:** Summary of failure mechanisms of SIBs under low-temperature conditions.

Category	Key Factors	Observed Phenomena/Issues	Consequences
**Electrolyte**	Increased viscosity	Reduced ionic conductivity; slower ion transport; electrolyte freezing/salt precipitation	Difficult desolvation process; degraded battery kinetics; performance deterioration
**Cathode Materials**	Slowed ion diffusion	Reduced Na^+^ diffusion rate in cathode bulk	Compromised structural stability
**Anode Materials**	Interfacial instability	Sodium metal deposition; unstable solid electrolyte interphase (SEI) film	Disrupted electrical contact; performance degradation
**Electrode/Electrolyte Interface**	Reduced compatibility	Poor electrolyte/electrode compatibility; increased charge transfer resistance	Severe polarization during cycling; reduced efficiency/cycle stability; capacity fade
**Overall Failure Mechanism**	Coupled multi-factor effects	Blocked Na^+^ diffusion (bulk, electrolyte, SEI); inefficient interfacial charge transfer	Comprehensive kinetic decline; significant capacity/lifetime reduction

**Table 2:** A comparison table of polyanionic materials, layered transition metal oxides, and Prussian blue analogs at low-temperature conditions.

Performance Metrics	Polyanionic Materials	Layered Transition Metal Oxides	Prussian Blue Analogs
**Cycling Stability**	Na_3_MnTi(PO_4_)_3_@rGO-KB: 111.4 mAh/g at −30 °C; Na-rich Na_4_V_2_(PO_4_)_3_ full-cell: 55.1 mAh/g at −30 °C.	Modified LiCoO_2_ (Li-ion battery): 179.2 mAh/g at −25 °C, but low-temperature data for Na-ion TMOs are limited.	FeVO-PBA maintains high capacity across −60–80 °C (exact values unspecified); Ba^2+^-embedded PBA: 83.41 mAh/g at 6C (temperature unspecified).
**Rate Performance**	Na_3_MnTi(PO_4_)_3_@rGO-KB: 85.3% capacity retention after 1000 cycles at −30 °C; K/Co co-doped NVP: 70.41% retention after 500 cycles.	Modified LiCoO_2_: 91% capacity retention after 300 cycles at −25 °C.	FeVO-PBA: stable over 30,000 cycles; Ba^2+^-embedded PBA: 96.6% retention after 150 cycles.
**Structural Stability**	NMTP@rGO-KB exhibits high-rate capability at −30 °C (specific values unspecified).	No data on low-temperature rate performance for Na-ion TMOs.	FeVO-PBA: 56.1 mAh/g at 100C; Ba^2+^-embedded PBA: 83.41 mAh/g at 6C
**Energy Density**	High stability due to NASICON framework; enhanced by carbon coating/doping.	Prone to interfacial side reactions and cracks; stabilized by surface coatings.	Open 3D framework may retain crystal water; stabilized by cation intercalation.
**Energy Density**	Relatively low energy density.	High energy density (e.g., LiCoO_2_: >250 Wh/kg).	FeVO-PBA full-cell: 259.7 Wh/kg.
**Low-Temperature Adaptability**	Modified NVP operates at −50–80 °C.	Modified TMOs perform well at −25 °C, but Na-ion system data are limited.	PBAs function across −60–80 °C.

**Table 3:** Key additives in this section.

Additive Category	Specific Name	Role/Mechanism	Performance Enhancement	Research Team
**Solvent-based additives**	CaCl_2_	Reduce the freezing point of the electrolyte and improve ionic conductivity at low temperatures.	Ionic conductivity of 7.13 mS cm^−1^ at −50 °C; capacity retention of 86.7% after 1000 cycles of full battery at −30 °C.	Zhu [129]
**Film-forming additives**	ES	Optimization of Na^+^ solvation structure to reduce desolvation energy and interfacial impedance.	Capacity retention of 88.2% for 200 cycles at 0.1 C.	Zhong [130]
**Film-forming additives**	FEC + Sn(OTf)_2_	Catalyzes the PC ring-opening reaction and enhances sodium ion migration.	QSPE ionic conductivity 0.42 mS cm^−1^; cycling stability of soft pack battery at −20 °C.	Yang [131]
**Conductivity-enhancing additives**	MeTHF	Induced anion/π-dominated solvation structures stabilize ion transport channels.	Coulomb efficiencies exceed 99% at −25 °C and −40 °C.	Ge [132]
**Stabilizing additives**	HFT + LiNO_3_	Synergistic dissolution of LiNO_3_ to form a Li_3_N/LiF-rich SEI layer.	Enhanced low-temperature interfacial stability of lithium systems.	Jang [133]
**Stabilizing additives**	[Li(15-crown-5)]NO_3_	In situ generation of highly ion-conducting Li_3_N interfacial layers.	No capacity degradation for 250 cycles of Li||LiCoO_2_ cells at −20 °C.	He [134]
**Stabilizing additives**	PQA-NO_3_	Cations build inorganic SEIs, and anions form low-solvation structures.	At −85 °C, a soft-pack battery retains 48.1% room temperature capacity; 3.0 C-multiplier high-rate discharge at −50 °C.	Zhang [135]

**Table 4:** Compare the strategies in this paper in terms of key parameters such as initial capacity, capacity retention, and operating temperature.

Strategy Category	Specific Approach	Initial Capacity (mAh g^−1^)	Capacity Retention	Operating Temperature (°C)	Cycle Life (Cycles)	References
**Electrolyte Optimization**						
**Solvent Regulation**	Methyl propionate (MP)-FEC electrolyte	109.6 (0.1C)	89%	−25	500	Liu et al. [33]
	THF-TPP mixed ether electrolyte	N/A	94.1%	−40	100	Yin et al. [47]
**Sodium Salt Engineering**	NaTFPB additive in diglyme	N/A	N/A	−20	N/A	Hu et al. [115]
**Additives**	Ethylene sulfate in carbonate electrolyte	N/A	88.2%	−40	200	Zhong’s team [130]
**Cathode Materials**						
**Polyanionic (NASICON)**	K-doped Na_3_V_2_(PO_4_)_3_ (NVP-K_0.05_)	72 (2C)	N/A	−25	N/A	Shen et al. [64]
**Layered Oxide**	P2-type Na_0.696_Ni_0.329_Mn_0.671_O_2_	N/A	N/A	−30	N/A	Liu et al. [68]
**Prussian Blue Analogs**	Co_0.7_Ni_0.3_-PBA	109	N/A	−30	N/A	Zhang et al. [73]
**Anode Materials**						
**Hard Carbon (HC)**	Zn-doped HC	443	85%	−40	400	Lu et al. [78]
**Alloy-Based**	Sb@graphene composites	265	63.7%	−20	100	Huang et al. [84]
**Conversion-Type**	FeS@graphitic carbon	311	N/A	−25	80	Fan et al. [92]
**Interface Engineering**						
**SEI/CEI Modification**	Na_2_Se/V heterointerface layer	N/A	86.5%	−40	700	Xia et al. [151]
	Fluorinated cyclophosphazene (PFPN) additive	N/A	~85%	0	450	Zhang et al. [98]
**Solvation Structure**	Weakly solvated ether (MeTHF) electrolyte	243.2 (HC)	N/A	−60	N/A	Fang et al. [107]

## Data Availability

Data are contained within the article.
